# Organelle Sorting and Proteomic Analysis to Identify Proteins Involved in the Uptake and Intracellular Trafficking of Nanoparticles

**DOI:** 10.1002/smtd.202502004

**Published:** 2025-12-17

**Authors:** Hector Garcia Romeu, Anna Salvati

**Affiliations:** ^1^ Department of Nanomedicine and Drug Targeting Groningen Research Institute of Pharmacy University of Groningen Groningen 9713 AV The Netherlands

**Keywords:** endocytosis, fluorescence assisted cell sorting, intracellular trafficking, nanomedicine, nanoparticle receptors, nanoparticles, organelle sorting, proteomics

## Abstract

Monitoring nanoparticle uptake and intracellular trafficking by cells is required to understand how nanomedicines are processed by cells. Many methods currently applied to achieve this test the involvement of known mechanisms and pathways after blocking them, or by measuring colocalization of nanoparticles with known intracellular compartments. However, unknown and non‐canonical mechanisms are often involved in nanomedicine uptake and the organelles in which they are trafficked are not fully identified. Therefore novel methods to characterize how and where nanomedicines are internalized and trafficked in unperturbed cells are highly sought. Here a novel platform is presented to characterize uptake and intracellular trafficking of nanoparticles without requiring prior knowledge on the involved mechanisms and their intracellular location. After uptake and cell lysis, organelle sorting by fluorescence assisted cell sorting is used to isolate and purify the organelles in which nanoparticles are internalized and trafficked. Next, the composition of the recovered organelles with nanoparticles is determined by organelle proteomics. Using polystyrene nanoparticles to validate the method, it is shown that this workflow allows to discover novel receptors involved in nanoparticle uptake and proteins mediating nanoparticle trafficking toward the lysosomes. The knowledge gained is essential to test and improve novel formulations for nanomedicine applications.

## Graphical abstract

A new method to identify proteins involved in the uptake and intracellular trafficking of nanoparticles is presented. After nanoparticle uptake, cells are lysed and the organelles with nanoparticles are purified using fluorescence assisted cell sorting. Next, their composition is determined by proteomic analysis. The method allows to characterize cellular pathways in unperturbed cells and without requiring prior knowledge.

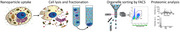

## 1 Introduction

Nano‐sized materials can be used for targeted drug delivery thanks to their ability to exploit cellular mechanisms to enter cells. In order to fully exploit their potential, it is necessary to gain a better understanding of the biological mechanisms by which they are internalized and trafficked by cells.^[^ ^1^, ^2^, ^3^, ^4^, ^5^, ^6^ ^]^ Many studies focus on phenotypic screenings of panels of candidate proteins to determine their involvement in the uptake and trafficking of nano‐sized objects.^[^ ^5^, ^7^, ^8^, ^9^, ^10^ ^]^ Although these studies offer important insights on the role of known endocytic proteins in nanoparticle uptake, methods that allow to identify all of the proteins involved, including potential novel proteins not yet associated to nanoparticle uptake and intracellular trafficking are required.^[^ ^11^ ^]^ To this end, here we apply a platform based on organelle flow cytometry coupled to organelle sorting and proteomic analysis for the purification and characterization of the subcellular compartments in which nanoparticles are internalized and trafficked.^[^ ^12^ ^]^

Many methods have been developed for cell fractionation and the purification of specific organelles known to be involved in endocytosis.^[^ ^13^, ^14^, ^15^ ^]^ However, these methods do not allow the *de novo* discovery of novel trafficking compartments not yet characterized. By taking advantage of the unique properties of nanoparticles, instead, novel methods to isolate all the organelles in which nanoparticles are found can be developed, without pre‐knowledge or pre‐assumptions on their identity. For instance, magnetic nanoparticles have been used to recover all organelles in which they are distributed following internalization by cells.^[^ ^16^, ^17^, ^18^, ^19^ ^]^ However, such approaches are fully dependant on the magnetic properties of the nanomaterial, which limits applications, especially when considering typical nanomedicines. On the other hand, nanomedicines are routinely tagged with fluorescent probes for visualization and tracing, thus methods using fluorescence to identify and isolate all organelles in which they are trafficked may find easier application. Within this context, in the last years, it has become increasingly possible to use flow cytometry to detect sub‐cellular structures down to ≈100 nm.^[^ ^20^ ^]^ This has been used to characterize different organelles, such as endosomes^[^ ^21^ ^]^ and lysosomes,^[^ ^22^ ^]^ and it is routinely applied for the characterization of exosomes and extracellular vesicles.^[^ ^20^, ^23^, ^24^ ^]^ Recently, organelle flow cytometry has been used to gain information on the intracellular location of nanoparticles and in this way determine their intracellular trafficking kinetics.^[^ ^12^ ^]^ At the same time, first particle flow cytometers have been developed, which are specifically built for the detection and characterization of sub‐micron, sub‐cellular objects. Similarly, the latest generations of fluorescence activated cell sorters (FACS) have enabled the sorting of nano‐sized vesicles such as secretory granules and are increasingly applied for the isolation and separation of exosomes.^[^ ^25^, ^26^ ^]^

Within this context, here we show that fluorescent nanoparticles can be used to detect and isolate by FACS all the organelles in which they are internalized and trafficked for subsequent characterization of their composition by proteomics. In fact, thanks to their high degree of labelling, fluorescent nanomaterials can be purified using standard sorters readily available in many laboratories for cell isolation (as opposed to specialized vesicle sorters). Cells were incubated with 100 nm fluorescently‐labelled carboxylated polystyrene nanoparticles, used as a model system to set up the method and showcase its potential. Then all organelles with nanoparticles have been isolated by FACS. Purification by FACS allowed subsequent analysis by proteomics of the sorted organelles with nanoparticles in order to characterize their composition. This workflow allowed us to identify many proteins involved in nanoparticle uptake and intracellullar trafficking. The identified targets have been validated by RNA interference and their role characterized. The presented approach can be easily translated to other fluorescent nanomaterials and fluorescently labelled nanomedicines in order to gain new understanding on the molecular details of nanoparticle uptake and intracellular trafficking by cells, thus ultimately contributing to the improvement of nanomedicine targeting and efficacy.

## 2 Results

### 2.1 Isolation and Purification of the Organelles Containing Nanoparticles

**Figure 1:**
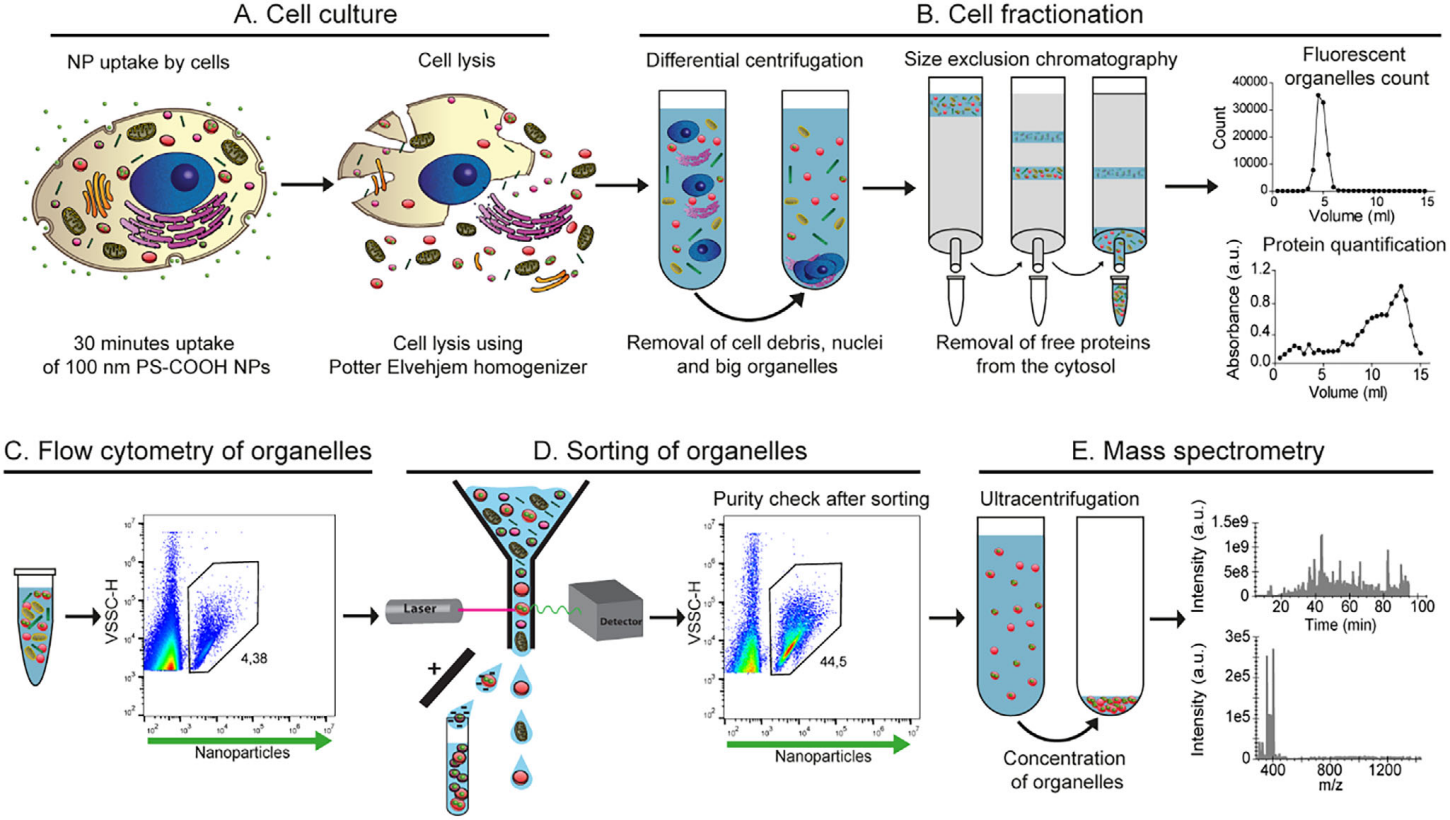
Scheme of the method used in this study to isolate the organelles involved in the internalization and intracellular trafficking of nanoparticles for proteomics characterization. A) Hela cells are incubated with fluorescent 100 nm PS‐COOH nanoparticles (30 min). After that, the cells are lysed in order to extract all organelles. B) Differential centrifugation is performed to remove the biggest organelles, such as nuclei and cell debris. The supernatant containing the smaller organelles is loaded on a size exclusion chromatography column to remove the free proteins from the cytosol. Each fraction eluting from the column is collected, and measured by high‐sensitivity flow cytometry to identify the fractions in which all cell organelles elute. C) Protein quantification of each fraction confirms separation of free cytosolic proteins eluting at later fractions. (C) Example of a flow cytometry dot plot of violet side scattering (VSSC) and nanoparticle fluorescence (green fluorescence) of the organelles. The population of organelles containing the fluorescent nanoparticles can be easily separated from the rest of the organelles. D) All organelle fractions are sorted by flow cytometry assisted cell sorting in order to purify the organelles containing fluorescent nanoparticles. High‐sensitivity flow cytometry of the sorted organelles confirms increased purity after sorting (roughly 10x). E) Finally, the sorted organelles are concentrated by ultracentrifugation and digested for characterization by mass spectrometry. Image created using Adobe Illustrator.

In order to set up the method, human cancer epithelial HeLa cells were used as a standard cell line routinely applied both in the nanomedicine field to study nanoparticle uptake, and in the endocytosis field, for large and genome‐wide screening of uptake and trafficking pathways.^[^ ^5^, ^6^, ^8^, ^9^, ^11^, ^20^, ^27^ ^]^ Yellow‐green fluorescently labeled carboxylated polystyrene (PS‐COOH) nanoparticles of 100 nm diameter were selected as well‐characterized model nanoparticles with high labeling and low photobleaching, for which extensive information on their interactions with cells is already available.^[^ ^7^, ^27^, ^28^, ^29^ ^]^ Additionally, these nanoparticles are known to be stable and form homogeneous dispersions also in the culture medium with serum used for cell studies.^[^ ^12^ ^]^ Thus, cells were incubated for 30 min with the nanoparticles, after which they were lysed using a Potter homogenizer following previously optimized protocols (**Figure** **1**).^[^ ^12^ ^]^ Next, some fractionation steps were followed to increase the purity of the sample prior to sorting (Figure 1B). More in detail, differential centrifugation was used to remove cell debris and nuclei, and size exclusion chromatography (SEC) was used to remove the free cytosolic proteins, which would confuse the characterization of the organelle proteome. All organelle fractions eluting from the column were collected. We previously showed that by using this procedure for cell lysis and fractionation, intact organelles can be recovered, hence excluding potential loss of organelle content.^[^ ^12^ ^]^ The organelles containing the fluorescent nanoparticles could be easily detected by flow cytometry using high‐sensitivity settings (Figure 1C; Figure S1, see Supporting Information for details on setting optimization). When measuring the same organelle samples using standard FACS for cell sorting, which often do not offer similar sensitivity, the organelles containing nanoparticles could still be detected, but they could not be fully separated from the background of the empty organelles (also in Figure S1, Supporting Information). Nevertheless, by selecting the channels with highest sensitivity for organelle and nanoparticle detection and by optimizing the speed of the sorting, which affects the purity of the sorted sample (see Figures S2 and S3, Supporting Information for more details), the organelle containing nanoparticles could be sorted. High‐sensitivity organelle flow cytometry of the sorted samples confirmed that even though non‐fluorescent events were still detected, sorting allowed to increase the fraction of the organelle‐containing nanoparticles of roughly 10 times (Figure 1C,D; Figure S4, Supporting Information).

Due to the low amount of proteins contained in a single organelle compared to a full cell, in order to achieve enough protein for proteomics analysis, a total of ≈40 million fluorescent organelles were sorted in each experiment in ≈40 mL. Different procedures were tested to concentrate the sorted organelles for proteomics (Table S1 and Figure S5, Supporting Information). The best results in terms of purity and reproducibility were obtained using ultracentrifugation to pellet the organelles and Rapigest surfactant to recover them. Finally, the samples were analysed by mass spectrometry (Figure 1E).

### 2.2 Proteomic Characterization of the Organelles Involved in Nanoparticle Uptake and Early‐Trafficking

**Figure 2:**
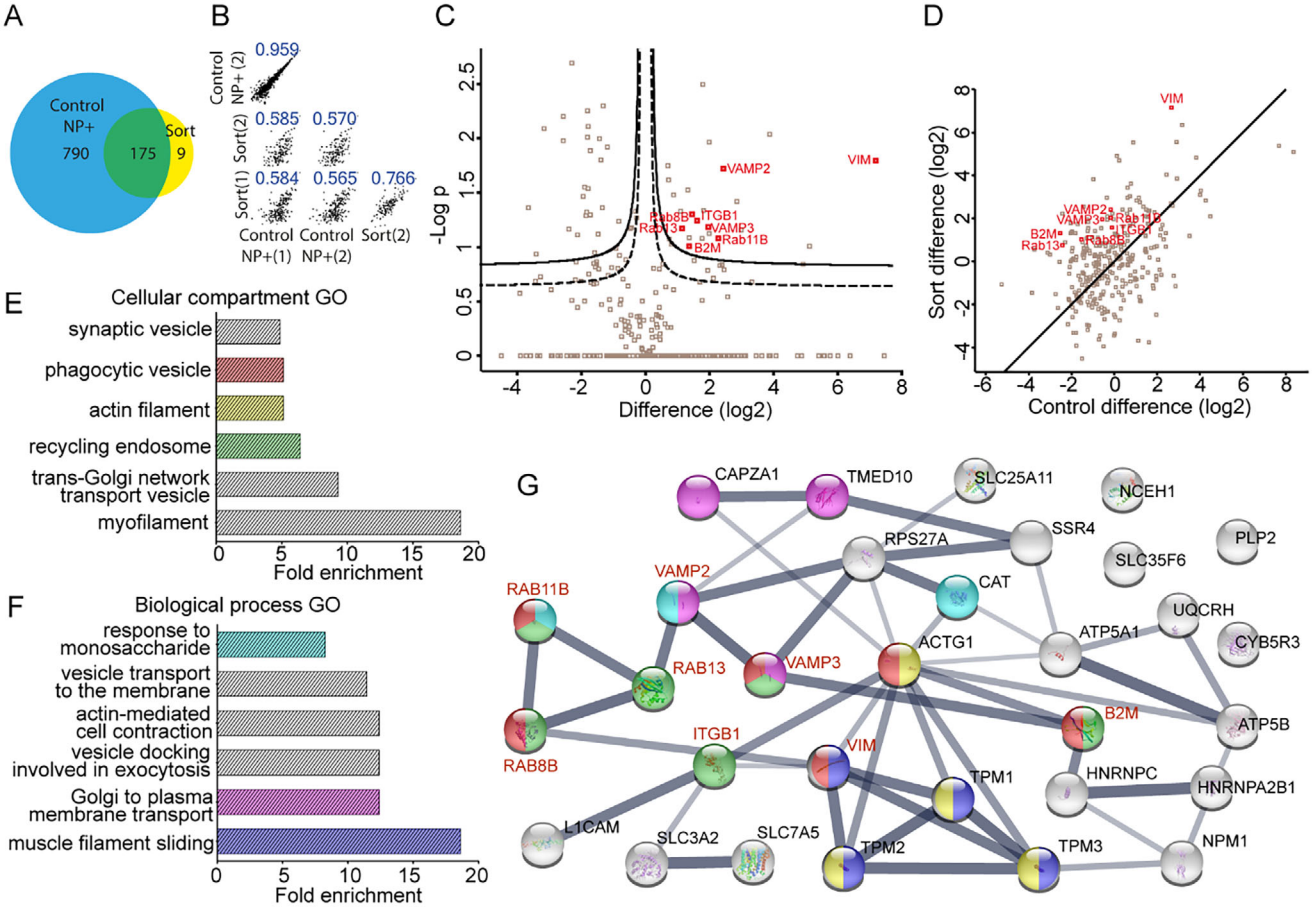
Proteomic characterization of the organelles involved in uptake and intracellular trafficking of nanoparticles. A) Venn diagram comparing the total number of proteins identified in the organelles before (control NP^+^) and after sorting (sort). B) Scatter plot comparing the abundance (iBAQ) of the proteins identified in the different samples. Two replicate samples were analyzed for each condition (*n* = 2). In blue, the Pearson correlation coefficient is shown for each comparison. C) Volcano plot to compare protein abundance in the sorted organelles in respect to the organelles before sorting. A *t*‐test was performed to determine the significance of the difference in iBAQ of the proteins. The dotted line corresponds to a 0.05 FDR and the solid line to a 0.01 FDR. On the right side of the volcano, several proteins enriched after sorting could be identified. D) Scatter plot comparing the protein enrichment of the sorted organelles in respect to the unsorted organelles from cells incubated with nanoparticles (*Y* axis, Sort difference), with the enrichment of unsorted organelles from cells incubated with nanoparticles in respect to the organelles from untreated cells (*X* axis, Control difference). The regression line shows the points where the enrichment was the same for both comparisons (x = y). E,F) Gene ontology (GO) analysis of the proteins enriched after sorting (from panel C), showing the statistically overrepresented cellular compartments (E) and biological process (F) gene ontology (GO) groups. The gene ontology groups were sorted for fold enrichment and a threshold of minimum 3 proteins identified per group and *p*<0.01 was applied. A Fisher's exact test was performed to determine the p‐values. G) STRING network analysis of the significantly enriched proteins from panel C. The line thickness corresponds to the confidence strength of the protein interaction. The same colors are applied for some of the groups identified in the GO results (E,F) and the proteins in those groups shown in the network analysis (G). A panel of proteins with highest enrichment and strongest interactions (whose names are written in red in C,D,G) were selected for further validation.

In order to take into account the presence of residual non‐fluorescent events in the purified sorted sample (Figure 1D), the organelles recovered after fractionation before sorting were also analysed. Thus, the protein composition of the organelles before and after sorting was compared using label‐free intensity based absolute quantification (iBAQ) (**Figure** **2**).^[^ ^30^ ^]^ As expected, a much higher amount of proteins was identified in the control unsorted organelle sample. Importantly, these included almost all of the proteins identified in the sorted organelles (Figure 2A), consistent with their purification. Scatter plots of the abundance of all identified proteins (Figure 2B) showed high correlation between replicate samples, confirming good reproducibility, but slightly lower for the sorted organelles. This is probably due to their lower protein content. Importantly, the correlation between controls and the sorted samples was much lower, which again is consistent with their purification. The volcano plot in Figure 2C shows the significantly depleted (left) and enriched (right) proteins in the sorted sample in comparisons to the unsorted organelle control. A total of 35 proteins were significantly enriched (false discovery rate, FDR<0.05) (see Supporting Information file for complete results).

As an additional control, the composition of the unsorted organelles was compared to that of organelles recovered in the same way from untreated cells that were not incubated with nanoparticles (Figure S6, Supporting Information). This allowed to determine eventual changes in protein content upon incubation with the nanoparticles. Many unique proteins were identified in the organelles from cells incubated with nanoparticles, and gene ontology (GO) analysis showed interesting enriched GO groups, such as “transferrin transport” and “late‐endosome membrane.” Additionally, several of the common proteins were enriched upon incubation with the nanoparticles, including proteins involved in the positive regulation of early‐endosome to late endosome. Because of this, we compared the proteins of the sorted organelles with those of the unsorted organelles from untreated cells (Figure 1D). For most proteins, the enrichment observed upon sorting was higher than the enrichment observed (in unsorted organelles) upon incubation with the nanoparticles (left side of the regression line x = y). This suggested that the enrichment observed in the sorted sample was not solely a consequence of cell exposure to nanoparticles, but rather due to the purification of the organelles with nanoparticles achieved with sorting.

We then performed GO analysis on the 35 enriched proteins identified in the sorted organelles (Figure 2E,F). A high enrichment of myofilaments and biological processes connected to the cell cytoskeleton was observed, due to several tropomyosins being identified. The cell cytoskeleton controls the sorting and movements of intracellular organelles, thus it is likely involved also in the trafficking of the organelles containing nanoparticles.^[^ ^31^ ^]^ Although a better understanding of how intracellular movements of organelles are orchestrated via such components is also of interest, in order to gain insight on the organelles in which nanoparticles were found, we focused on the other classes of proteins identified. Many of the enriched GO groups were related to uptake (phagocytic vesicle) and intracellular trafficking (trans‐Golgi network transport‐vesicle and recycling endosome). Similarly, in relation to biological processes (Figure 2F), the enriched GO groups included Golgi to plasma membrane transport, vesicle docking involved in exocytosis and vesicle transport to the plasma membrane.

When looking at the identity of the proteins in these categories, different cell membrane proteins and receptors were identified, including integrin beta‐1 (ITGB1), beta‐2 microglobulin (B2M) and cell adhesion molecule L1 (L1CAM), and two members of the Solute Carrier Family (SLC3A2 and SLC7A5), whose complex was found to be involved in the entry of hepatitis C virus.^[^ ^32^ ^]^ Many proteins known to be involved in intracellular trafficking and receptor recycling were also enriched, such as different Rab (Ras‐related in brain) proteins (Rab8B, Rab11B, Rab13), and vesicle‐associated membrane proteins (VAMPs) VAMP2 and VAMP3. Other interesting targets included the transmembrane P24 trafficking protein TMED10, which recently was found to have a role in the entry of vaccinia virus and uptake of large unilamellar vesicles.^[^ ^33^ ^]^ Next, a String analysis was performed to visualize the networks of the enriched proteins (Figure 2G) and showed some degree of interaction between most of the identified proteins. Importantly, the proteins with strongest interactions were also all enriched with high significance (FDR<0.01, solid line). Altogether, this confirmed that the method allowed to isolate and characterize the organelles involved in uptake and intracellular trafficking of nanoparticles.

As a further example, the organelles recovered from cells incubated for longer times with the nanoparticles, i.e., 2 h, where extracted and sorted in the same way and their composition compared to those sorted after 30 min. After longer incubation time, it is expected that more nanoparticles have already reached the lysosomes, where usually they are trafficked after uptake.^[^ ^7^, ^28^, ^34^ ^]^ However, the results showed that the samples were very similar both in relation to the identified proteins and their abundance (iBAQ), thus hardly any protein was significantly enriched (Figure S7, Supporting Information). When comparing each sample to the unsorted organelle control, the enriched proteins were the same, but a higher enrichment was observed in the 30 min sample (left side of the regression line x = y). The same was observed when looking at the abundance (iBAQ) of all the endosomal proteins identified (GO cellular compartment endosome). This suggested that, despite the different incubation time, nanoparticles were found in comparable compartments already after 30 min and after 2 h. This was also a further confirmation of the reproducibility of the different steps of organelle recovery and purification. Of note, the experimental design can be easily adapted, should one aim to distinguish and characterize the different compartments in which nanoparticles are trafficked at different times after uptake (as opposed to continuous incubation, when nanoparticles are found in all compartments along the trafficking process): this can be done by first letting the nanoparticles enter cells for a short time and then washing away the extracellular nanoparticles and extracting the organelles with nanoparticles at different times after nanoparticle removal (so called “pulse and chase”).

Similarly, the method can be easily transferred to other cells (including in hard‐to‐transfect cells where methods based on genetic manipulations to characterize cellular pathways cannot be easily applied) and nanoparticles, provided that their fluorescence is strong enough for detection. To confirm this, in Figure S8 (Supporting Information), we show the enrichment obtained via organelle sorting by FACS from human TRP3 liver sinusoidal cells and THP1 monocytes incubated with the same 100 nm polystyrene, as well as from TRP3 cells incubated with 200 nm polystyrene. By recovering organelles with nanoparticles for different nanoparticle types and/or cell types as in these examples, the method allows to study how nanoparticle uptake and intracellular trafficking change with nanoparticle properties (in this example, nanoparticle size) and in different cell types.

### 2.3 Validation of the Proteins Enriched After Sorting

**Figure 3:**
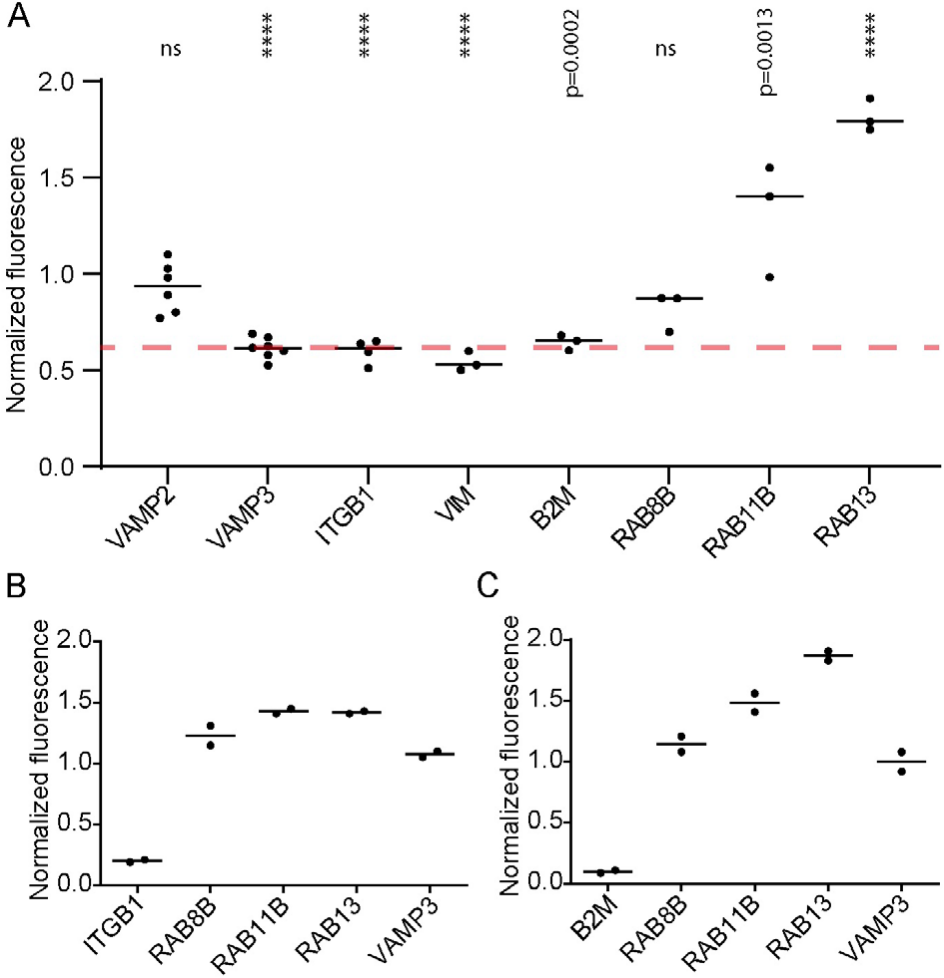
Effect of identified targets on nanoparticle uptake and membrane expressionof cell receptors. A) Nanoparticle uptake in HeLa cells after silencing the expression of some of the proteins identified by proteomic anlaysis. Briefly, the expression of a panel of proteins identified by organelle proteomic was silenced in HeLa cells. Next, the silenced cells were incubated for 1 h with 100 µg mL^−1^ 100 nm PS‐COOH nanoparticles in complete cell culture medium. The results are the median cell fluorescence obtained by flow cytometry in the silenced cells, normalized by the fluorescence of HeLa cells silenced with a scramble RNA as a control. A minimum of three independent experiments (up to 7) with three replicate samples each were performed and their average is indicated with a solid line (*n* = 3–7). A dashed red line at 60% uptake is included as a reference. An unpaired two‐sided one‐way analysis of variance (ANOVA) with Dunnett's correction for multiple comparisons was used for statistical analysis (*p* values are reported when *p* < 0.05, which was considered significant; ^****^ *p* < 0.0001). B,C) Normalized fluorescence of the membrane expression of ITGB1 (B) or B2M (C). Briefly, after silencing the expression of the indicated targets, HeLa cells were immunostained against ITGB1 (B) or B2M (C) in order to determine effect of silencing on their membrane expression. The results of two independent experiments are shown, each including three replicate samples (*n* = 2). The solid lines indicate the average of the replicated experiments.

Organelle sorting and proteomic analysis allowed to identify several enriched proteins. As a following step, in order to confirm the role of the identified proteins in uptake and intracellular trafficking, we selected a panel of eight targets among those with highest enrichment and stronger interactions for further validation. These included the receptor integring beta‐1, ITGB1, and membrane protein beta‐2 microglobulin, B2M, together with the three identified Rab proteins (Rab8B, Rab11B and Rab13), as well as VAMP2 and VAMP3. In order to validate the role of the identified targets in nanoparticle uptake, first, RNA interference was used to silence their expression in HeLa cells. RT‐PCR (Reverse transcription polymerase chain reaction) confirmed excellent silencing efficacy with >75% reduction in expression for all targets (Figure S9, Supporting Information). Then, the effect of silencing on nanoparticle uptake was determined (**Figure** **3**).

#### 2.3.1 Membrane Proteins and Potential Nanoparticle Receptors

As shown in Figure 3A, uptake was strongly reduced (≥40%) after silencing ITGB1 and B2M, suggesting a role of these cell membrane proteins as nanoparticle receptors. Identifying the receptors involved in nanoparticle uptake is an essential step for nanomedicine targeting. Involvement of integrins in nanoparticle uptake has been previously reported and several strategies for integrin targeting are being investigated, also known their increased expression in several diseases, such as cancer and in inflamed endothelium.^[^ ^35^, ^36^, ^37^, ^38^ ^]^ Instead, to the best of our knowledge, this is the first observation on the involvement of B2M in nanoparticle uptake. B2M is part of the major histocompatibility complex class I (MHC I), which usually displays peptides derived from the intracellular degradation of proteins to CD8^+^ T cells in order to activate an immune response. Further studies are required to understand its role in nanoparticle uptake and eventual implications for the immune response.

Roughly 50% uptake reduction was observed after silencing vimentin expression. Vimentin, which was the protein with highest enrichment in the sorted organelles (fold increase >143 times, see Supporting file) is a filament protein, that is also known to have different roles in intracellular trafficking, including in the recycling of integrin beta 1.^[^ ^39^, ^40^ ^]^ Interestingly, increasing reports have showed that vimentin can also be expressed on the cell membrane, and many examples of a role of vimentin in the uptake of several types of viruses are emerging, including recently for SARS‐Cov2.^[^ ^41^, ^42^, ^43^, ^44^ ^]^ The reduced uptake observed after silencing vimentin suggests a similar role of this protein for nanoparticle uptake.

At a broader level, the identification of multiple cell membrane proteins affecting uptake shows that nanoparticle can engage with multiple receptors in the same cells, as also suggested by other studies.^[^ ^11^, ^35^, ^45^ ^]^ It would be interesting to determine whether nanoparticles interact with these different membrane proteins via molecules in their corona, as observed in other cases.

#### 2.3.2 Role of Rab11B and Rab13 in Uptake and Receptor Recycling

Opposite to what observed when silencing ITGB1, B2M, and VIM, silencing Rab11B and Rab13 expression strongly increased nanoparticle uptake (roughly 50% and 80% respectively), while no effects were observed after silencing Rab8b.

Rab11B and Rab13 are known to be involved in endosome recycling and exocytosis. The increased fluorescence after silencing would be consistent with a reduced recycling and/or exocytosis of the internalized nanoparticles, a highly debated topic in the nanomedicine community.^[^ ^46^, ^47^, ^48^ ^]^ Alternatively, the increased uptake could be explained by changes in membrane expression of proteins and receptors involved in nanoparticle uptake, such as B2M and ITGB1. For instance, it was previously reported that Rab11B and Rab13 are also involved in the recycling of ITGB1.^[^ ^49^, ^50^ ^]^ A similar role in (ADP‐ribosylation factor 6) ARF6‐mediated ITGB1 recycling was reported also for VAMP3.^[^ ^51^ ^]^ However silencing VAMP3 reduced uptake of around 40% (while no effects were observed after silencing its isoform VAMP2).

Thus, in order to test eventual effects on receptor expression, we quantified mRNA levels and cell membrane expression of ITGB1 and B2M (Figure 3B,C respectively) in cells silenced for the 3 Rab proteins and VAMP3. RT‐PCR showed that silencing these targets did not affect the mRNA levels of ITGB1 and B2M (Figure S10, Supporting Information). Consistent with the observed reduction in gene expression (Figure S9, Supporting Information), quantification of ITGB1 and B2M on the cell membrane confirmed that silencing strongly reduced their membrane expression (roughly 80% and 90% reduction, respectively). No changes were observed after silencing VAMP3 and only a minor increase in cells silenced for Rab8B. Instead, in cells silenced for Rab11B and Rab13, ITGB1 membrane expression increased of around 40% in both cases (Figure 3B) and B2M was increased of 50 and 90% respectively (Figure 3C). The strong increase in membrane expression of ITGB1 and B2M likely explains the increased uptake in cells silenced for Rab11B and Rab13. Thus, these Rab proteins have a clear role in the recycling of at least some of the receptors involved in the uptake of these nanoparticles. Further research is needed to determine whether in addition to this, exocytosis of nanoparticles may also be present.

#### 2.3.3 Role of VAMP3 in the Early‐Trafficking of the Nanoparticles

**Figure 4:**
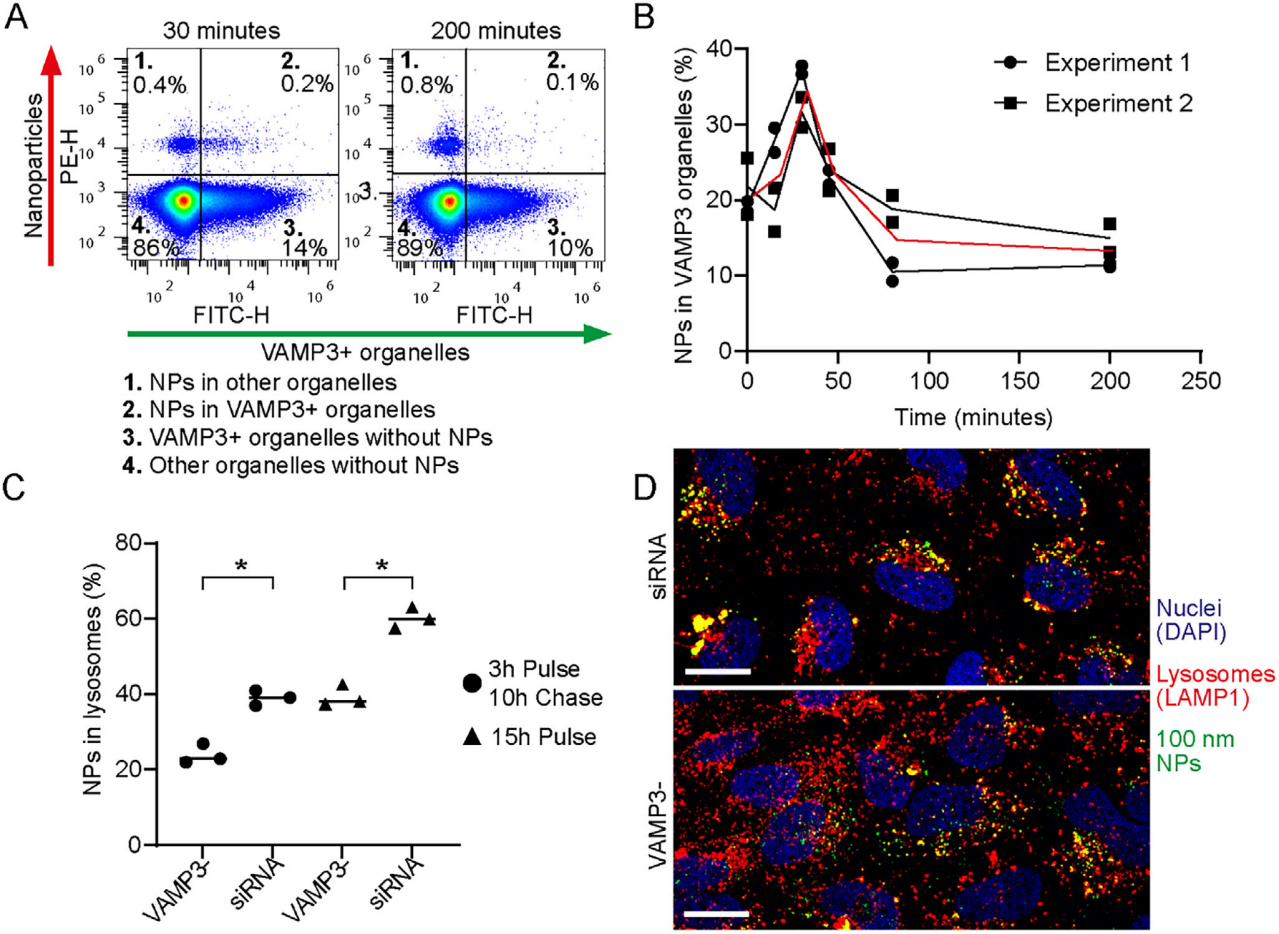
VAMP3 role in nanoparticle uptake and intracellular trafficking in HeLa cells. A,B) Colocalization of 100 nm PS‐COOH nanoparticles with VAMP3^+^ organelles. Briefly, HeLa cells were transfected with a construct to express eGFP‐labelled VAMP3 and incubated with 100 µg mL^−1^ red 100 nm PS‐COOH nanoparticles for 20 min, then cells were washed and the organelles were extracted at different times after nanoparticle removal (0, 15, 30, 45, 80, and 200 min) as described in the Methods. The dot plot (A) shows the fluorescence of the nanoparticles (PE channel) vs the fluorescence of the VAMP3^+^ organelles, 30 and 200 min after nanoparticle removal. A total of four populations can be distinguished in each dot plot and their fraction is indicated. In quadrant 2, the fraction of VAMP3^+^ organelles with nanoparticles can be obtained. The plot in B shows the colocalization values obtained in this way from organelles extracted at different times after incubation with the nanoparticles, corrected for the transfection efficiency of each independent experiment (see Methods and Figure S12, Supporting Information for details, raw data and transfection efficiency). The results of two independent experiments are shown, each with two replicate samples for each time point, together with a black line passing through their average. The red line indicates the average between the independent experiments (*n* = 2). C) Fraction of nanoparticles in the lysosomes after silencing VAMP3. Briefly, HeLa cells were silenced for VAMP3, then they were incubated with 100 µg mL^−1^ PS‐COOH for 3 h (pulse), followed by nanoparticle removal and10 h chase, or to 25 µg mL^−1^ for 15 h. Then the organelles were extracted and lysosome stained for organelle flow cytometr as described in the Methods. In this way, the fraction of nanoparticles in the lysosomes after silencing VAMP3 expression could be obtained and compared to the results in cells silenced for a scramble RNA as a control (see Figure S13, Supporting Information for full results). The results of three replicate samples are shown together with a solid line indicating their average (*n* = 3). A *t*‐test was performed to compare the colocalization values and *p*<0.01 is indicated with an asterisk. D) Confocal microscopy images of HeLa cells incubated with 25 µg mL^−1^ 100 nm PS‐COOH (green) for 3 h, followed by nanoparticle removal and 10 h chase. Red: LAMP1 (Lysosomal‐associated membrane protein 1) immunostained lysosomes. Blue: DAPI (4',6‐diamidino‐2‐phenylindole) stained nuclei. Scale bar: 20 µm. On the top, an image of cells silenced for VAMP3 is shown and on the bottom, cells silenced with a scramble RNA as a control.

We then investigated in more detail the role of VAMP3 in nanoparticle uptake and intracellular trafficking (**Figure** **4**). Vesicle‐associated membrane proteins (VAMPs) are a family of SNARE (soluble NSF attachment protein receptor) proteins involved in different trafficking processes from and to the cell membrane, via both clathrin‐dependent and –independent processes.^[^ ^52^ ^]^ For instance, Shiga toxin promotes clathrin‐independent uptake of VAMP2 and VAMP3 and it has been reported that VAMP3 is involved in the recycling of ITGB1, as well.^[^ ^52^, ^53^ ^]^ In order to test potential effects after longer incubation with the nanoparticles, uptake kinetics were determined up to 30 h (Figure S11, Supporting Information). Over longer incubation time, a mild reduction in uptake was observed also after silencing VAMP2, while the observed effect of VAMP3 increased further.

In order to confirm nanoparticle colocalization with VAMP3‐positive organelles, HeLa cells were transfected with a construct to express eGFP‐labelled VAMP3. Thus, nanoparticles were added to the cells for 15 min followed by nanoparticle removal and further growth in nanoparticle‐free medium, and at different times after nanoparticle removal, cells were lysed and organelles collected for organelle flow cytometry. The organelles containing nanoparticles could be easily detected (quadrant 1 and 2 in the double scatter plots of Figure 4A), as also the VAMP3‐positive organelles (quadrant 2 and 3). In this way, the presence of a fraction of nanoparticles in VAMP3‐positive organelles could be confirmed (quadrant 2). By extracting organelles at different times after incubation with nanoparticles, the kinetics of nanoparticle transit into VAMP3‐positive compartments could be determined as well. The results showed increasing colocalization in the first 30 min (up to 33%), later decreasing to ≈10% (Figure 4C, after correction for the transfection efficacy, as explained in more detail in Figure S12, Supporting Information). Using organelle flow cytometry in the same way, we previously showed that these nanoparticles first transit in (Early endosome antigen 1) EEA1‐positive compartments, i.e., early endosomes, and later arrive to LAMP1‐positive organelles, i.e., lysosomes.^[^ ^12^ ^]^ The overlap of the corresponding colocalization kinetics (here reproduced from ref.[12] in Figure S13, Supporting Information) shows that nanoparticles first reach the early endosomes, then are found in compartments associated with VAMP‐3 and finally are trafficked into the lysosomes.

Consistent with this, it has been reported that silencing VAMP3 perturbs the accumulation of Uukuniemi virus (UUKV) to the lysosomes.^[^ ^54^ ^]^ In order to test similar effects for nanoparticle trafficking, we determined accumulation of the nanoparticles in the lysosomes after silencing VAMP3 expression. Confocal fluorescence microscopy suggested that after silencing VAMP3 more nanoparticles were distributed throughout the cell, and not yet arrived in the lysosomes (also in Figure 4A). In line with this, quantification by organelle flow cytometry confirmed that nanoparticle colocalization with the lysosomes was lower in VAMP3‐silenced cells (Figure 4D, including results from cells incubated with nanoparticles at a lower concentration, and corresponding flow cytometry dot plots in Figure S14, Supporting Information).

## 3 Conclusion

In this work, we developed an approach based on organelle sorting by flow cytometry and organelle proteomics to recover all organelles in which nanoparticles are distributed following uptake and determine their composition. The method relies on the use of fluorescently labeled nanomaterials, thus can find many applications for nanomedicine characterization, including in cells difficult to manipulate and transfect for similar intracellular trafficking studies. This approach allows to gain new insights on nanoparticle uptake and intracellular trafficking, without prior knowledge or hypothesis on the pathways involved. Thus, we used polystyrene nanoparticles as a model to illustrate the potential of this method: indeed, despite extensive information is already available on the uptake and intracellular trafficking of polystyrene nanoparticles, organelle sorting and proteomic analysis allowed us to identify multiple nanoparticle receptors, including novel receptors that – to the best of our knowledge ‐ were not yet associated with polystyrene nanoparticle uptake, such as ITGB1 and B2M, as well as proteins controlling their recycling, thus affecting their membrane expression. Additionally, the method allowed us to discover that VAMP3 is involved in the uptake and intracellular trafficking of these nanoparticles. By using organelle flow cytometry to quantify nanoparticle colocalization, we confirmed that after uptake and trafficiking in early endosomes, nanoparticles indeed transit in VAMP3‐compartments and VAMP3 also controls the subsequent nanoparticle trafficking toward the lysosomes.

These results illustrate the potential of the approach presented in identifying novel targets involved in nanoparticle uptake and intracellular trafficking and resolving molecular details of the processes involved. These are important details which need to be addressed to improve our understanding on how cells process these materials for their application in nanomedicine.

## 4 Experimental Section

### Cell Culture

HeLa cells (ATCC CCL‐2) were grown in complete cell culture medium (cMEM) composed by Eagle's minimum essential medium (MEM) (Gibco, Waltham, MA, USA) supplemented with 10% v/v fetal bovine serum (FBS, Gibco). The cells were maintained under standard conditions (37 °C, 5% CO_2_). All the experiments were performed using cells between passages 3 and 20 after defrosting. Cells were tested for mycoplasma once per month to exclude contamination.

### Nanoparticles

FluoSpheres carboxylated polystyrene (PS‐COOH) nanoparticles of 100 and 200 nm diameter fluorescently labeled in green (maximum excitation 505 nm/ emission 515 nm) and red (580/605) were purchased from Invitrogen (Life Technologies, Carlsbad, CA, USA). These nanoparticles were known to form stable dispersions also in the cell culture medium supplemented with serum used for incubation with cells.^[^ ^12^ ^]^

### High‐Sensitivity Organelle Flow Cytometry

A Cytoflex flow cytometer model S (Beckman Coulter, Pasadena, CA) equipped with four lasers (405, 488, 561, 605 nm) was used to analyse the samples and check oragenelle enrichment before and after sorting. A detailed explanation and optimization of the settings to measure organelles was described in Garcia‐Romeu et al.^[^ ^12^ ^]^ In brief, a threshold was applied in the Violet Side Scattering height (VSSC‐H) at 1500. Next, PBS was run for 5 min to assess the amount of background events. A maximum of 200 events per second (eps) was allowed in the instrument at slow flow rate (10 µL min^−1^). The gain in the VSSC channel was decreased if higher eps were detected. Next, a Megamix mixture was used to calibrate the instrument. The Megamix mixture was prepared by adding the Megamix‐Plus FSC and the Megamix‐Plus SSC (catalogue numbers 7802 and 7803, Biocytex, Marseille, France) in a 1:1 ratio and final volume of 200 µL. The Megamix mixture consists of a total seven populations beads with sizes ranging between 100 and 900 nm (100, 160, 200, 240, 300, 500, and 900 nm). These beads are fluorescent in the FITC channel. If those populations were not accurately separated in the VSSC‐H vs FITC‐H dot plot, the gains in those channels were tuned until the 7 populations could be detected. At this point the instrument was set for high‐sensitivity measurements of organelles. Unless specified otherwise, all the samples were measured for a total 30 µL at slow flow rate (10 µL min^−1^) at a concentration between 1000 and 3000 eps. Most of the times samples were diluted to reach this range of eps. When the sample was diluted, the dilution factor was annotated in the file of the sample. Data was acquired using CytExpert 2.0 software (Beckman Coulter) and analyzed using Flowjo software version 10.7.1 (BD Biosciences, San Jose, CA).

### Organelles Extraction for Sorting

HeLa cells were cultured in T175 flasks with 1.5 × 10^7^ cells for 24 h prior to the experiment. The next day the cells were incubated with the yellow‐green fluorescent PS‐COOH nanoparticles at a concentration of 100 µg mL^−1^ for 30 min in complete cell culture medium supplemented with serum (cMEM). Complete medium with serum was used to avoid starvation which could affect uptake and intracellular trafficking. After that, the cells were washed with 20 mL cMEM three times and 20 mL PBS five times to remove any leftover nanoparticles adsorbed on the cells. Next, the cells were harvested by adding 4 mL Trypsin‐EDTA at 37 °C for 5 min. The trypsin activity was stopped by adding 12 mL cMEM, and the cells were recovered and centrifuged (200 g, 5 min). The pelleted cells were resuspended in 5 mL PBS and centrifuged again (200 g, 5 min). From this step onward, all the steps were performed at 4 °C. The cells were resuspended in 2 mL lysis buffer, consisting of 5 mm Tris‐Base, 1 mm EDTA supplemented with a tablet of protease inhibitor cocktail (Sigma–Aldrich St Luis, USA) (1 tablet for 15 mL of lysis buffer). Roughly 1 mL of lysis buffer per each ten million cells was used. The cells were lysed using a Potter‐Elvehjem homogenizer to extract the organelles. In order to avoid damaging the organelles, the total lysis percentage in the cells was always measured by counting the cells stained by Trypan blue in a hematocytomer before and after using the Potter‐Elvehjem homogenizer. All the experiments were performed in the regime between 60 and 80% lysis percentage, which usually took ≈20 strokes in the Potter‐Elvehjem homogenizer. After that, the cells were centrifuged (1500 g, 10 min) to remove the cells debris and nuclei. The supernatant was transferred to a clean Eppendorf and the pellet was discarded. Finally, the free proteins from the cytosol were removed by size exclusion chromatography in a 10 cm and 15 mL Sepharose CL‐4B gravity column (10273151, Sigma–Aldrich St Luis, USA), as previously optimized.^[^ ^12^ ^]^ The organelle extract was added at the top of the column. After all the sample entered the column, PBS was added and 0.5 mL fractions eluting from the column were collected. A total of 15 fractions was collected in each experiment. The organelles containing nanoparticles eluted between the fractions 6 and 9, as confirmed by flow cytometry of each of the recovered fractions.^[^ ^12^ ^]^ Then, the collected organelle fractions were sorted in order to purify the organelles containing the nanoparticles. Organelle extraction and sorting were always performed on the same day.

### Measuring and Sorting Fluorescent Organelles by FACS

The extracted organelles were sorted at the Flow Cytometry Unit of the University Medical Centre of Groningen. A MoFlo Astrios (Beckman Coulter) equipped with equipped with seven lasers (355, 405, 488, 532, 561, 592, and 640 nm) was used to sort the organelles. Prior to each session, the instrument was calibrated using standard procedures for cell sorting in order to set appropriate gains in each channel. For organelle sorting the gain settings of the fluorescence channels were not modified. For the detection of the organelles, two different set ups were used, with a threshold on either Side Scattering (SSC) or FITC intensity. For the Side Scattering Threshold set up, the threshold was applied in the 561 nm SSC channel, which was determined to be the most accurate for the detection of the Megamix mixture beads populations (Figure S1, Supporting Information). A control sample consisting of an unsorted organelle extract was used each time to determine the SSC gates for sorting. For the FITC threshold set up, the threshold was applied in the FITC‐H channel. The FITC‐H threshold was established in each sorting session by measuring an organelle sample which did not contain fluorescence nanoparticles. No gating was required in the FITC‐H set up; all the events with FITC fluorescence higher than the threshold were sorted. Increasing the gain voltages for the channels of interest was tested, but it did not improve detection. Next, the samples were measured and sorted at a speed of 1500–2000 eps approximately. The sorting was always performed at ≈0.3 psi difference between the pressure of the instrument and the pressure of the sample. Approximately 1 million events were sorted in each experiment, while for sample preparation for proteomic analysis this was increased to up to a total of 40 million events. After sorting, the purity of the sorted sample was determined by high sensitivity organelle flow cytometry using the Cytoflex S flow cytometer and the sorted organelles were stored at 4 °C overnight prior to sample preparation for proteomics. Data was acquired using the Summit v62 software (Beckman Coulter) and analysed using Flowjo software version 10.7.1 (BD Biosciences, San Jose, CA).

### Sample Concentration After Sorting

After sorting, the sample was concentrated using a Sorvall Ultracentrifuge Discovery 90SE (Kendro Laboratory Products, Newtown, Connecticut, U.S.A) with a swinging bucket rotor SW32‐Ti. Approximately 40 mL samples were obtained after sorting. This volume was divided in two thin‐wall polypropylene tubes (Beckman Coulter) which were weighted and equilibrated. Next, the sample was ultracentrifuged at 25 000 rpm (80 000 g) for 1 h. The supernatant was discarded, and the pellet, which was not visible, was resuspended by adding 50 µL RapiGest SF (Waters, UK) 0.2% in ABC prewarmed at 95 °C in each tube. In order to ensure recovery of all pelleted organelles, the RapiGest solution was left in the tube for 2 h at room temperature, covering the tubes to avoid contamination. Finally, the sample was recovered by thoroughly pipetting around the bottom of the tube, and the sample was collected in low‐binding tubes. After this step, the sample could be stored at −20 °C until preparation for proteomics.

### Sample Preparation for Proteomics

RapiGest SF (Waters) was used as a surfactant for the sample preparation for proteomics following the manufacturer´s instructions. In brief, after storage at −20 °C, the sample was boiled at 95 °C for 5 min to help denaturalize hydrophobic proteins. After bringing the sample to room temperature, dithiothreitol (DTT) was added to the sample to a final concentration of 10 mm and incubated at 60 °C for 30 min. Next, iodoacetamide (IAA) was added to the sample to a final concentration of 15 mm and incubated in the dark for 30 min. Finally, trypsin was added for digestion at 1:40 w/w if the amount of protein was known. The protein concentration in the samples obtained from the sorting was too low to be determined. In those samples, enough trypsin was added to digest 1 µg of protein. Next, the samples were incubated at 37 °C overnight for enzymatic digestion. The next day, formic acid (FA) was added to the digested protein samples to stop the trypsin digestion. The final FA concentration was kept at ≈0.1% (pH <*2*). (Note: It is important to use high purity FA). The sample was incubated at 37 °C for 30–45 min. Slight cloudiness could be observed since the hydrolytic RapiGest SF by‐products were water immiscible. The acid treated samples were centrifuged at 20 000 g for 30 min for two times to remove the nanoparticles and the precipitated RapiGest SF. The supernatants were then carefully transferred to another low‐binding Eppendorf tube. Finally, the samples were concentrated by spin drying the sample in a Speedvacuum concentrator (Vacufuge Plus, Eppendorf) and resuspended in 15 µL 0.1% FA (which is the minimum volume allowed for injection in the mass spectrometer). After that, the samples were injected in the mass spectrometer on the same day or stored at −20 °C.

### Mass Spectrometry Analysis

After sample preparation, NanoLC‐MS/MS injection was performed on a Q Exactive Plus hybrid quadrupole− orbitrap mass spectrometer (ThermoFisher) equipped with an UltiMate 3000 RSLCnano system (ThermoScientific). The peptides were loaded into a trap column (Acclaim PepMap 100 C18 LC columns, ThermoFisher) and then separated into an analytical column (PepMap RSLC C18 columns, ThermoFisher). To separate peptides, a flow rate of 300 nL min^−1^ and a column temperature of 40 °C were used. Mass spectrometry data was measured using a data‐dependent top ten method.

### Database Searching and Quantification of the Proteomics Data

MS raw data was analysed using the software MaxQuant (version 1.6.5.0). Peptide and protein database search was performed against the human SwissProt database (UP000005640, 20380 entries, downloaded January 2021). The parameters for identification were set for a false rate discovery (FDR) of the proteins of 1% and a minimum of one razor or unique peptide per identification. Oxidation of methionine residues and acetylation of protein N‐terminus were selected as variable modifications, and carbamidomethylation on cysteine residues as fixed modification. Relative protein quantification was performed using the iBAQ algorithm in MaxQuant,^[^ ^30^ ^]^ considering only the proteins with a minimum of one unique peptide. The iBAQ values were analysed with the Perseus software (version 1.6.5.0). First, the proteins that were present in the contaminants database were discarded. The MaxQuant contaminant database was used to filter out contaminants in all the samples. In addition, the HeLa proteome (http://mapofthecell.biochem.mpg.de)^[^ ^55^ ^]^ was used to filter out any protein not present in the proteome. Next, each sample was normalized by the sum of the their total iBAQ values to account for the uncertainty in the amount of sample injected for the sorted samples. Volcano plots were shown with an FDR of 0.01 and 0.05. A gene ontology (GO) overrepresentation test was performed at http://pantherdb.org, where the significantly enriched proteins in the volcano plot were compared to the human genome or the protein list of a control. The GO overrepresentation test was performed with a Benjamin analysis, using a threshold of minimum 3 proteins per group and *p*‐value<0.01. The network analysis was performed at https://string‐db.org/cgi for the significantly enriched proteins in the volcano plot too.

### Plasmid Transfection

The constructs to express eGFP labelled vesicle associated membrane protein 3, eGFP‐VAMP3, was provided by Thierry Galli (Addgene plasmid # 42310 ; http://n2t.net/addgene:42310 ; RRID:Addgene_42310).^[^ ^56^ ^]^ Hela cells were transfected using the FuGENE HD transfection reagent (Promega) following the manufacturer's instructions. In brief, 50 000 cells per well were plated in 12‐wells 24 h prior to the transfection. A total of 400 ng of plasmid DNA per sample was used in the transfection, which was previously mixed with the FuGENE reagent at 3:1 ratio (3 µL of reagent per µg of DNA). The mixture was incubated for 15 min at room temperature and added to the wells. 48 h after transfection, red (580/605) 100 nm PS‐COOH nanoparticles were added to the transfected cells at 100 µg mL^−1^ concentration in cMEM for 30 min. Then, the cells were washed with fresh cMEM (1 mL) three times and PBS (1 mL) five times, to remove nanoparticles left in the wells. Next, to allow intracellular trafficking of the nanoparticles, fresh cMEM was added and cells further grown prior to organelle extraction after 0, 15, 30, 45, 80, and 200 min chase. The organelles were extracted as described in the previous sections. For each sample, prior to cell lysis, part of the cells were measured by flow cytometry to determine the cell transfection efficiency. Controls samples of cells without labelled VAMP3 and/ or without nanoparticles were also prepared to set the gates in the flow cytometer. Two independent experiments were performed, each containing duplicates for all the samples.

### RNA Interference in HeLa Cells

In order to silence the expression of the targets of interest (B2M, ITGB1, Rab8B, Rab11B, Rab13, VAMP2, VAMP3, and VIM), Oligofectamine transfection reagent (ThermoFisher) and Silencer Select siRNA (ThermoFisher) were used. In brief, Hela cells were plated at 13 000 cells density in 24‐wells plates 24 h prior to the silencing. The next day, the cells were washed with serum free MEM for 30 min. Meanwhile, 10 pmol of the siRNA was mixed with 1 µL oligofectamine per well in Opti‐MEM (ThermoFisher) following the manufacturer's instructions. The mixture was incubated for 20 min at room temperature before it was added to the wells in fresh serum free medium. Scrambled RNA (Negative 1 siRNA) was used as a control. Then, cells were incubated for 4 h at 37 °C. After that, 30% FBS MEM was added to the wells to bring them to standard cMEM conditions (10% FBS MEM). Cells were further grown for 72 h at 37 °C and 5% CO_2_.

After 72 h silencing, HeLa cells were incubated with 100 µg mL^−1^ 100 nm yellow‐green PS‐COOH nanoparticles for 1 h in cMEM. Cells were harvested with trypsin‐EDTA as previously described and measured at the Cytoflex S flow cytometer. The cell fluorescence of the silenced cells was normalized by the fluorescence of control cells silenced with scrambled siRNA in order to determine the effect of silencing on nanoparticle uptake.

### B2M and ITGB1 Membrane Expression After Silencing

In order to determine the membrane expression levels of B2M and ITGB1 after silencing their expression and in control cells silenced for scrambled siRNA, 70 000 cells per well were seeded in 6‐well plates. Then, silencing was performed as described above by using five times of all the volumes of reagents. After silencing, the cells were harvested using 1 mm EDTA. Then cells were spinned down (300 g, 5 min) and immunostained against B2M (Thermofischer, MA1‐19141) or ITGB1 (Abcam, ab24693) by resuspending the pellet in an antibody solution at 1:200 dilution for 1 h at 4 °C. Next, the residual unbound antibody was removed by centrifugation (300 g, 5 min) and resuspension in 1 mL PBS two times. Then, a secondary antibody against mouse IgG (Alexa Fluor 488 Goat Anti‐Mouse IgG, from Thermofischer, A‐11029) was added at 1:200 dilution for 1 h at 4 °C and cells were washed in the same way. After washing, cells were resuspended in 200 µL PBS and the samples were measured in the Cytoflex S flow cytometer. The results were normalized by the fluorescence of cells transfected with scrambled siRNA to determine the change in membrane expression.

### Nanoparticle Colocalization in the Lysosomes After Silencing VAMP3 Expression

HeLa cells were silenced for VAMP3 and with a scrambled siRNA as a control as described above. After silencing, cells were incubated with 100 µg mL^−1^ or 25 µg mL^−1^ 100 nm yellow‐green PS‐COOH nanoparticles for 3 h (pulse), then cells were washed and further grown in medium without nanoparticles (10 and 15 h chase, respectively). Next, the cells were harvested and organelles were extracted as previously described and immunostained against LAMP1 (Anti‐LAMP1−Cy3, from Sigma, L0419‐200UL). The antibody was added at a 1:200 dilution and incubated with the cells for 1 h at 4 °C. For the organelle samples, the free antibody was removed by SEC following the same procedure described for the removal of free cytosolic proteins. The eluted organelle fractions were measured at the Cytoflex S flow cytometer.

### mRNA Expression

The cellular expression levels of the silenced genes were determined by RT‐PCR. Cells were seeded in 24‐well plates and silenced as described above. Next, the total mRNA was extracted from the cells recovered from four replicate wells using a Maxwell 16 simplyRNA Cells Kit (Promega), following the manufacturer's instructions. cDNA was prepared in an Eppendorf Mastercycler gradient by reverse transcription from 0.2 µg RNA using a M‐MLV reverse transcripase (Promega) following manufacturer's instructions. The cycle which was followed was: 20  °C for 10 min, 42  °C for 30 min, 20  °C for 12 min, 99  °C for 5 min, and 20  °C for 5 min. Quantitative RT‐PCR was then used to quantify the gene expression levels (10 nm cDNA per sample, 4 replicate wells per target) using a SensiMix SYBR kit (Bioline) in a ABI7900HT sequence detection system (Applied Biosystems). The Ct values were obtained using SDS 2.4 software (Applied Biosystems). The average Ct value over the four replicate wells and its standard deviation were calculated. Results were expressed as fold‐change of the averaged Ct values obtained in untreated cells silenced with a scrambled siRNA (neg) in respect to the averaged Ct value of silenced cells (siRNA) as follows:

(1) $$Fold\;change=2^{-\left(Ct\;\left(neg\right)-Ct\left(siRNA\right)\right)}$$

### Immunostaining for Confocal Microscopy

For the microscopy experiments, a thin glass coverslip was added to bottom of the well in 24‐well plates. Cells were silenced for VAMP3 and a control scrambled siRNA as described above. After silencing, 100 nm yellow‐green PS‐COOH nanoparticles were added to the HeLa cells at 25 µg mL^−1^ in cMEM for 15 h. Next, the cells were washed with 1 mL cMEM and 1 mL PBS (2 times) and fixed with 200 µL 4% formaldehyde for 20 min in darkness. Next, the cells were permeabilized with 200 µL 0,1% Triton 100x for 5 min and washed with 1 mL PBS (2 times). LAMP1 antibody (BD Biosciences, 555798) was added at 1:100 dilution in 100 µL and incubated with cells for 1 h in darkness. The wells were washed with 1 mL PBS (2 times) and the secondary antibody (Alexa Fluor 647 goat anti‐rmouse IgG, from Thermofisher, A‐31573) was added at 1:200 dilution in 100 µL and incubated for 1 h in darkness. Finally, 100 µL DAPI 0.2 µg mL^−1^ was added for 5 min, washed with 1 mL PBS and the coverslip was mounted with MoWiol. Images were acquired using a Leica TCS SP8 confocal fluorescence microscope (Leica Microsystems), using a 405 nm laser for DAPI excitation, a 488 nm laser for the yellow‐green nanoparticles, and a 638 nm laser to detect the LAMP1 stained lysosomes.

### Statistical Analysis

The analysis performed to determine the proteins enriched after organelle sorting is described in the related section (Database Searching and Quantification of the Proteomics Data). In Figure 3A, the results are the median cell fluorescence obtained by flow cytometry in the silenced cells, normalized by the fluorescence of HeLa cells silenced with a scramble RNA as a control. A minimum of three independent experiments (up to 7) with three replicate samples each were performed and the results of individual experiments are shown together with their average (*n* = 3–7). An unpaired two‐sided one‐way analysis of variance (ANOVA) with Dunnett's correction for multiple comparisons was used for statistical analysis (*p* values are reported when *p* < 0.05, which was considered significant; ^****^ *p* < 0.0001). In Figure 3B,C, the results ITGB1 (B) or B2M (C) expression after silencing the different targets are normalized by the expression in untreated cells. The results of two independent experiments are shown, each including three replicate samples (*n* = 2). In Figure 4B, the colocalization values from organelles extracted at different times after incubation with the nanoparticles are shown, after normalization for the transfection efficiency of each experiment (See details in Figure S12, Supporting Information). The results of two independent experiments are shown, each with two replicate samples for each time point, together with a black line passing through their average. The red line indicates the average between the independent experiments (*n* = 2). In Figure 4C, the results of three replicate samples are shown together with a solid line indicating their average (*n* = 3). A *t*‐test was performed to compare the colocalization values and *p*<0.01 is indicated with an asterisk. Statistical analysis was performed using GraphPad Prism v.8 or higher.

## Conflict of Interest

The authors declare no conflict of interest.

## Supporting information



Supporting Information

Supporting Information

## Data Availability

The data that support the findings of this study are available from the corresponding author upon reasonable request.
