# Supplementary material for: Organelle Sorting and Proteomic Analysis to Identify Proteins Involved in the Uptake and Intracellular Trafficking of Nanoparticles
Source: Small Methods. 2025 Dec 17;10(3):e02004. doi: 10.1002/smtd.202502004 (PMC12893302; doi:10.1002/smtd.202502004)
Supplement: Supplementary file 1 — Supporting Information [file SMTD-10-e02004-s001.docx]

**Supporting Information**

*H. Garcia Romeu and A. Salvati*

**Supporting Methods**

**Organelle extraction and sorting from TRP3 and THP1 cells**

Acute monocytic leukemia cells (THP1 cells, ATCC TIB-202) were cultured in Roswell Park Memorial Institute 1640 medium (RPMI, ThermoFisher) supplemented with 10% FBS (ThermoFisher).

Human liver endothelial sinusoidal cells (TRP3 cells) were supplied by Birke Bartosch and Romain Parent.^[1]^ Cell culture flasks were previously coated with 0.1% gelatin diluted in cold PBS. TRP3 cells were cultured in MCDB131 medium (ThermoFisher) supplemented with 20% FBS (ThermoFisher), 50 µg/ml endothelial cell growth supplement (ECGS, Corning), 10 mM glutamine, 1µg/ml hydrocortisone (Sigma-Aldrich) and 250 µg/ml cAMP (Sigma-Aldrich). The cell medium was refreshed every 2-3 days.

All the cell lines were grown at 37°C and 5% CO_2_ and used between passages 3 and 20 after defrosting. The cells were tested for mycoplasma once per month to exclude contamination.

For organelle extraction and sorting, TRP3 cells were cultured in two T175 flasks with 5x10^6^ cells 48 hours prior to the experiment. The day of the experiment, the cells were washed once with serum-free MCDB prior to incubation with the nanoparticles. Yellow-green PS-COOH nanoparticles of 100 and 200 nm were dispersed to a final concentration of 100 and 200 µg/ml, respectively, in MCDB medium supplemented with 40 mg/ml human serum (HS, from pooled donors, from TCS Bioscience), in order to mimic more physiological serum concentrations. The nanoparticle dispersions were incubated with cells for 30 min. Next, cells were washed three times with cMEM (20 ml) and five times with PBS (20 ml), prior to cell lysis and organelle extraction, performed as described in the Methods.

For the THP-1 cell line, the day of the experiment 2x10^7^ cells were centrifuged (200 g, 5 min) and resuspended in serum-free RPMI (20 ml). A 100 nm yellow-green PS-COOH nanoparticle dispersion was prepared at 100 µg/ml in 40 ml RPMI supplemented with 40 mg/ml human serum (HS). Next, the THP-1 cells were centrifuged again and resuspended in the 40 ml nanoparticle dispersion and incubated with the nanoparticles in two T175 flasks for 30 min. After, cells were washed three times with cMEM (20 ml) and five times with PBS (20 ml) by centrifugation at 200 g for 5 min, followed by cell lysis and organelle extraction, performed as described in the Methods.

**Setting up the FACS to sort nano-sized objects**

Nowadays, some standard flow cytometers offer set ups that allow to measure nano-sized objects. This can be achieved in different ways, for instance, nano-sized objects can be detected by setting the instrument for high sensitivity using polystyrene FITC-labelled calibration nano-beads (Megamix beads) to set the voltage gains, and using a violet laser (405 nm) for side scattering detection (405-SSC) (instead of lasers at higher wavelength as commonly used), and lowering the threshold in this channel to the minimum value possible. **Supporting Figure S1A** shows an example of a measurement performed in this way with a high-sensitivity flow cytometer with a sample of Megamix beads, which consists of nanoparticles of 7 different populations in size (100, 160, 200, 240, 300, 500 and 900 nm). The same sample was tested on a standard cell sorter in order to select best settings for the sorting of nano-sized objects (Supporting Figure 1B-E). When we applied the same configuration as in the high-sensitivity flow cytometer (405-SSC threshold), only the 2 biggest populations (500 and 900 nm) were detected (Supporting Figure S1B). When we applied the threshold in the blue (488 nm) or yellow-red (561 nm) lasers SSC a total of 4 populations were detected (Supporting Figure S1B-C). Finally, we also tested the same sample using a threshold in the FITC-fluorescence channel (Supporting Figure 1D). This offered similar results, but only the fluorescent objects could be detected. The best results were obtained using the 561-SSC and the FITC-H threshold, even though also in these cases it was not possible to detect all the 7 populations, indicating overall a lower sensitivity. Next, we tested the quality of the sorting with the two different settings with a sample of organelles from HeLa cells incubated with 100 nm fluorescent PS-COOH nanoparticles. A control sample of organelles without nanoparticles was used to define the gate for the sorting of the fluorescent organelles (**Supporting Figure S2A**). Next, all the events inside the gate were sorted using the 561-SSC set up (Supporting Figure S2B). Even though, a population of fluorescent organelles could be detected, the population was not separated from the background and was cut by the SSC threshold. Instead, when using flow cytometry with high-sensitivity settings to measure the same sample before sorting (**Supporting Figure S3A**), thanks to the higher sensitivity, the population of fluorescent organelles was fully separated from the non-fluorescent organelles and fully included within the SSC threshold. When using the FITC-H threshold configuration for sorting (Supporting Figure S2C-D), the control sample with organelles without nanoparticles was used to set the threshold for the detection of fluorescent objects (Supporting Figure S2C). Next, the organelles from cells incubated with nanoparticles was measured and all the events detected were sorted (Supporting Figure S2D). With these settings, the gate used in the 561-SSC configuration only accounts for 36% of the fluorescent organelles detected. Thus, using the FITC-H threshold for detection allows to detect and sort a much higher number of fluorescent organelles in comparison to the 561-SSC threshold configuration.

The samples sorted with the 2 different settings were measured in the high-sensitivity flow cytometer to assess the purity of the sorting (Supporting Figure S3). First, the sample before sorting was measured to determine the percentage of fluorescent organelles (Supporting Figure S3A). With both settings, sorting resulted in approximately a 7-fold increase in purity in comparison to the unsorted sample (Supporting Figure S3B-C after sorting, in comparison to Supporting Figure S3A before sorting).

Next, the speed of the sorting was optimized. The samples in Supporting Figure S3B&C were both measured at 1500 events per second (eps). In the FITC-H configuration all the detected fluorescent events (all events shown in Supporting Figure S2D) were sorted, but in the 561-SSC configuration only 22% of the detected events were sorted (those shown in the gate of Supporting Figure S2B). The purity of the obtained sorted samples was 52% in the 561-SSC configuration and 60% in the FITC-H configuration (as it can be seen in Supporting Figure S3B and C, respectively). Instead, when increasing the speed of sorting in the FITC-H configuration to 5000 eps (Supporting Figure S3D), the purity of the sorting was slightly decreased (from 60% to 39%, as it can be seen in Supporting Figure S3C and D respectively). Moreover, we also noted that when using the 561-SSC configuration, the SSC of the sample was increased after the sorting because the events with low SSC could not be detected, thus they could not be sorted either (Supporting Figure S3A and B, respectively before and after the sorting). In other words, with these setting it was not possible to sort the smaller organelles with nanoparticles because their SSC was too low for SSC detection. On the contrary, these could be sorted when using the fluorescence of the nanoparticles for the threshold of detection, and as a reflection of this the SSC of the sample sorted in the FITC configuration remained the same before and after sorting (Supporting Figure S3A and S3B-C). Therefore, in order to be able to sort and include in our analysis the smaller organelles with nanoparticles, we used the FITC-H threshold for detection and in order to obtain a higher purity of the sorting, a lower speed of 1500 eps was used for sorting all samples. Despite the lower speed of sorting, given that by using the FITC-H threshold, 100% of the detected events were sorted, the time required for sorting was lower than when using the 561-SSC threshold configuration.

The dot plots in **Supporting Figure S4** show the two sorted samples used for proteomics characterization. Approximately a 9-10 fold increase in purity was achieved in two independent extractions and sorting experiments. This was estimated after correcting for the background events found in the PBS. Those events account for a small percentage of the sample when it is concentrated (for instance less than 5% when measuring at 2000-3000 eps).^[2]^ However, the background events can increase substantially when measuring a diluted sample (less than 1000 eps), such as the samples obtained after sorting. Because of this, a correction for background events was needed to determine the actual purity. This was done by measuring a sample only containing PBS and the sorted organelle sample using the same flow rate for the same volume. Then, the number of events detected in the PBS sample was subtracted from the total events in the organelle samples and the new percentages were calculated. The dot plots in Figure 1D indicate the percentage of events without correction, while the same dot plots are shown in Supporting Figure 4B with the percentages obtained after correction for the background events.

**Optimization of sample preparation for proteomic characterization**

Due to the low amount of protein contained in a single organelle compared to a single cell, a total of 40,000,000 organelles were sorted in each experiment in order to recover enough proteins for proteomic characterization. However, the samples were extremely diluted after sorting and 40,000,000 events we contained in approximately 40 ml volume. Thus, the sorted sample was concentrated by ultracentrifugation as described in the Methods. Then, different protocols were tested for sample preparation for proteomics. **Supporting Table S1**, and **Supporting Figure S5** show the results obtained when using different surfactants and methods to recover the organelle pellet after ultracentrifugation. High sensitivity flow cytometry was used to count the number of fluorescent organelles before and after the ultracentrifugation (see Supporting Table S1). The speed was high enough to pellet >90% events. However, with all methods tested, >50% of the fluorescent organelles could not be recovered (this was estimated by taking into account the volume of sample and number of fluorescent events detected before and after ultracentrifugation). Sodium dodecyl sulphate (SDS) 1% and Urea 8 M offered the best results (40% organelles recovered). Rapigest 0.2% also allowed recovery of roughly 25% of the sorted organelles. On the other hand, simple resuspension and mixing with PBS, a method usually used to recover exosomes after ultracentrifugation had a poor recovery (5%). The samples recovered with the different methods were also analysed by mass spectrometry in order to compare their protein content and the presence of contaminants such as keratins, which can constitute large part of the sample when protein content is particularly low (also in Supporting Table S1). Surprisingly, when using urea or SDS which had the highest organelle recovery, very few proteins were detected (<40 proteins) and a high percentage of contaminants was present in the samples (>90% of the total intensity). This result may be explained by the fact that when using these reagents solid-phase-extraction columns are required prior to trypsin digestion. These are likely to result in peptide loss. On the contrary, Rapigest which was not as efficient in the recovery of the organelles, did not require the use of solid-phase-extraction columns, and, likely because of this, it was the method that allowed to identify the largest amount of proteins. Based on all these results, we used Rapigest for the recovery of organelles after ultracentrifugation.


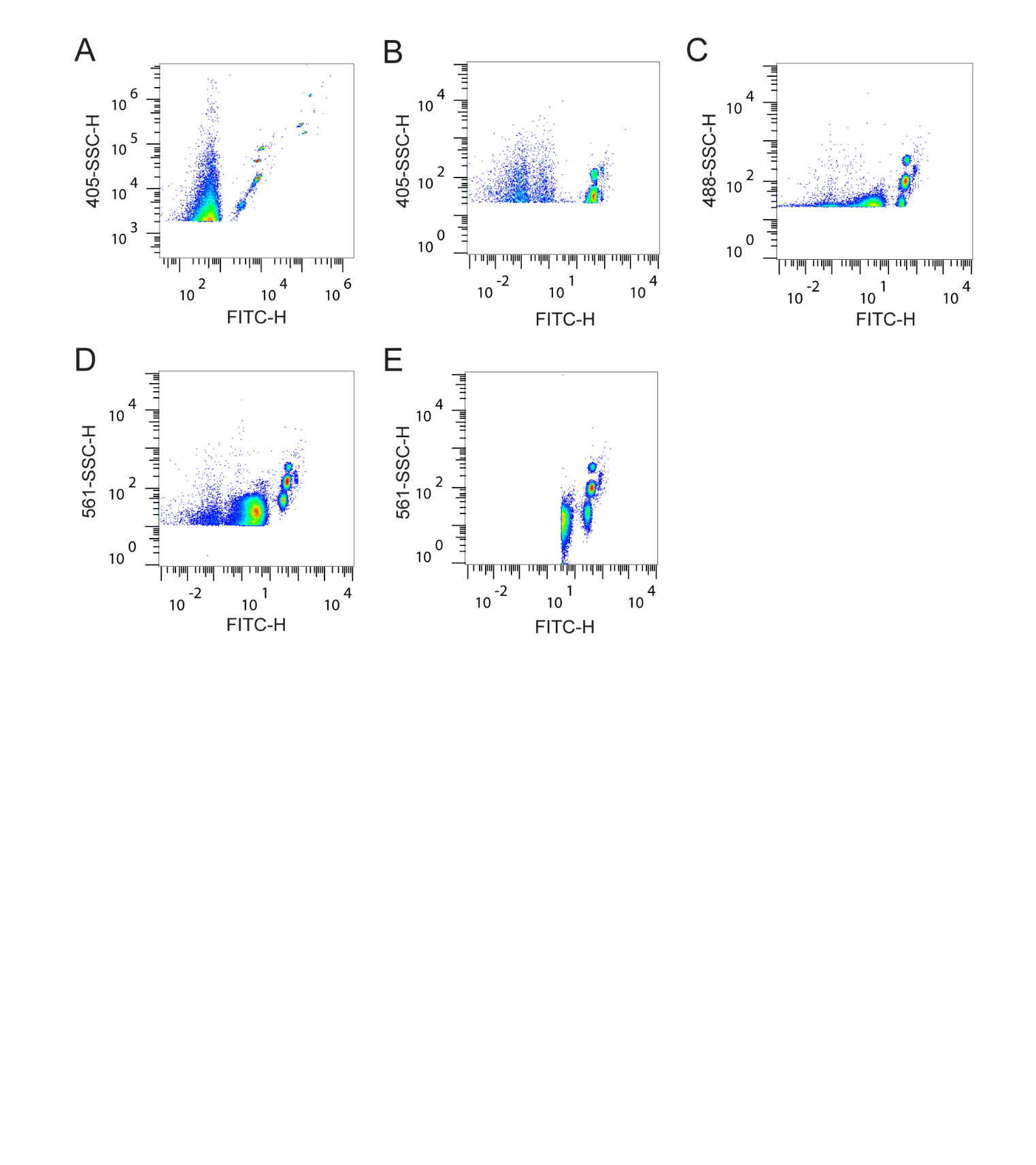


**Supporting Figure S1.** Optimization of FACS settings for the measurement of nano-sized objects. (A) Dot plot (405-VSSC vs FITC-H) of the results obtained with the Megamix calibration beads populations on a high-sensitivity flow cytometer (Cytoflex S). The results obtained on a high-sensitivity flow cytometer are shown here as a reference to allow comparison with the results obtained with the same sample on the FACS used for organelle sorting (B-E). (B-E) Dot plots of the measurements of the Megamix calibration beads (FITC-H channel) in the MoFlo Astrios sorter using different thresholds for detection. The thresholds were applied in the violet laser (405 nm) SSC-H (B), the blue laser (488 nm) SSC-H (C), the yellow-red laser (561 nm) SSC- H (D) and on the fluorescence in the FITC-H channel (E). The Megamix calibration beads were prepared by mixing the Megamix-Plus SSC and Megamix-Plus FSC reagents in a ratio 1:1 and final volume of 200 µl. The Megamix calibration beads consist of a mix of beads fluorescent in the FITC channel and different sizes: 100, 160, 200, 240, 300, 500 and 900 nm. With high-sensitivity flow cytometry (A), all the populations could be distinguished. In comparison when using a standard cell sorter, when the threshold was applied in the violet laser SSC-H (B), only two populations could be detected (500 and 900 nm). When the threshold was applied in the blue (C) or yellow-red (D) lasers SSC-H, three populations could be fully distinguished (300, 500 and 900 nm). Also, a fourth population was detected, but it was cut by the thresholds, especially when using the blue laser (C). A similar result was obtained when using the threshold in the FITC-H channel (E). Overall, the best results were obtained when using the threshold for detection in the yellow-red laser SSC-H (D) and the FITC-H channel (E). Thus, thresholds were applied in these channels to define the best settings for the sorting of cell organelles (see Supporting Figure S2).


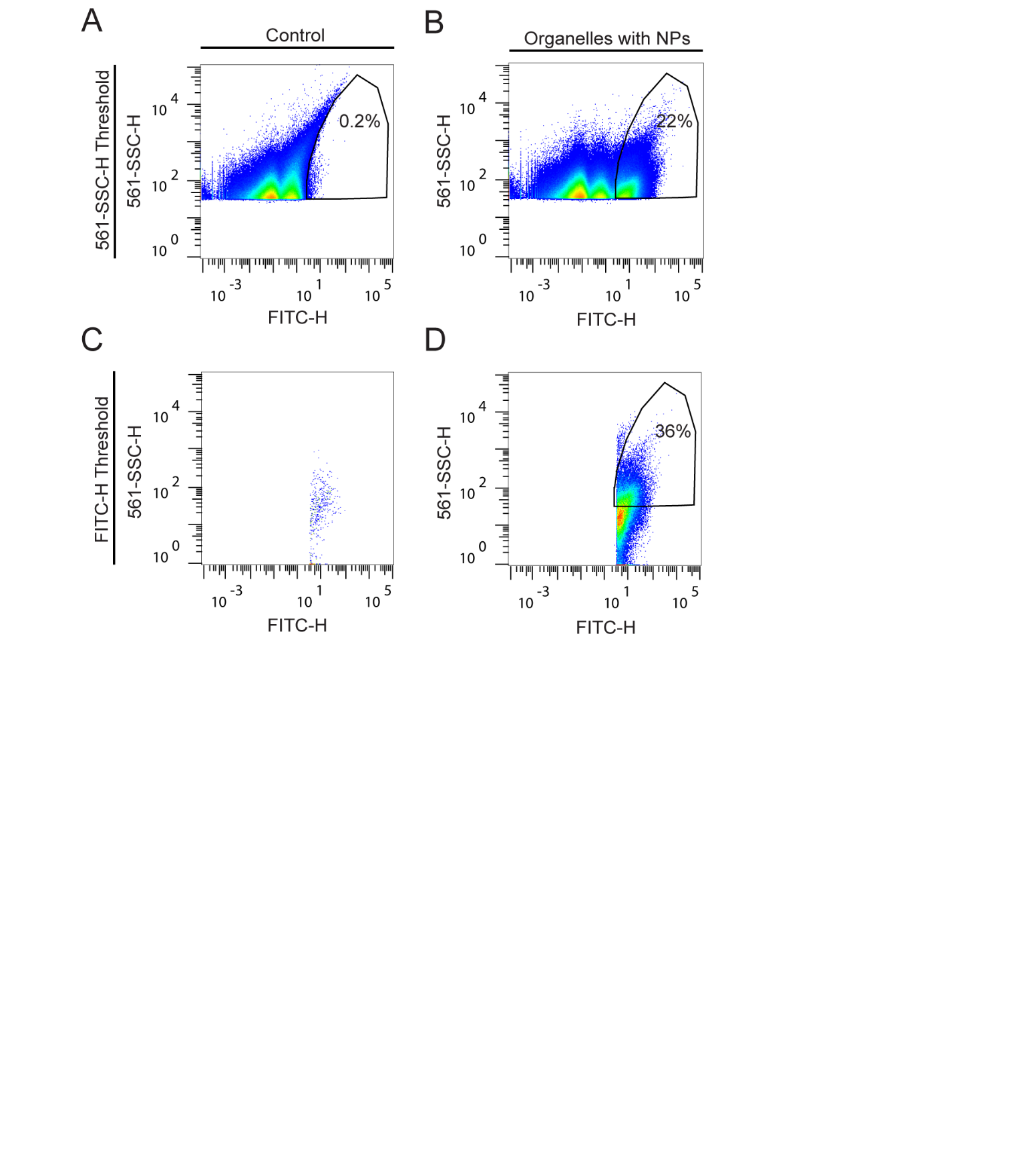


**Supporting Figure S2.** Detection of nano-sized objects by FACS. (A-D) Dot plots (561-SSC-H vs FITC-H) from organelles extracted from HeLa cells in a MoFlo Astrios FACS. Briefly, HeLa cells were incubated with yellow-green PS-COOH 100 nm nanoparticles for 30 minutes at 100 µg/ml and the organelles were extracted as described in Methods. (A-B) Dot plots of the results obtained when applying the threshold for detection in the yellow-red laser (561 nm) SSC-H channel, for (A) a control sample consisting of organelles from untreated cells without nanoparticles and (B) a sample of organelles with fluorescent nanoparticles (B). (C-D) Results for the same samples when applying the threshold for detection in the FITC-H channel. A control sample consisting of organelles without fluorescent nanoparticles was measured to set up the gate used for sorting, as shown in panels A and B, or to set up the fluorescent threshold, as shown in C. In the latter case, all the organelles that passed the threshold for detection in the FITC-H channel were sorted as shown in D. In panel D, the gate set for sorting when the threshold was applied in the yellow-red laser SSC-H is also included for comparison. The results showed that when the threshold was applied in the yellow-red laser SSC-H, only 36% of the fluorescent organelles that are detected when applying the FITC-H threshold were sorted, thus more than 60 % of the organelles containing nanoparticles could not be detected nor sorted, compared to the FITC-H threshold set up. Because of this, the sorting of the organelles with nanoparticles was performed using the FITC-H threshold set up.


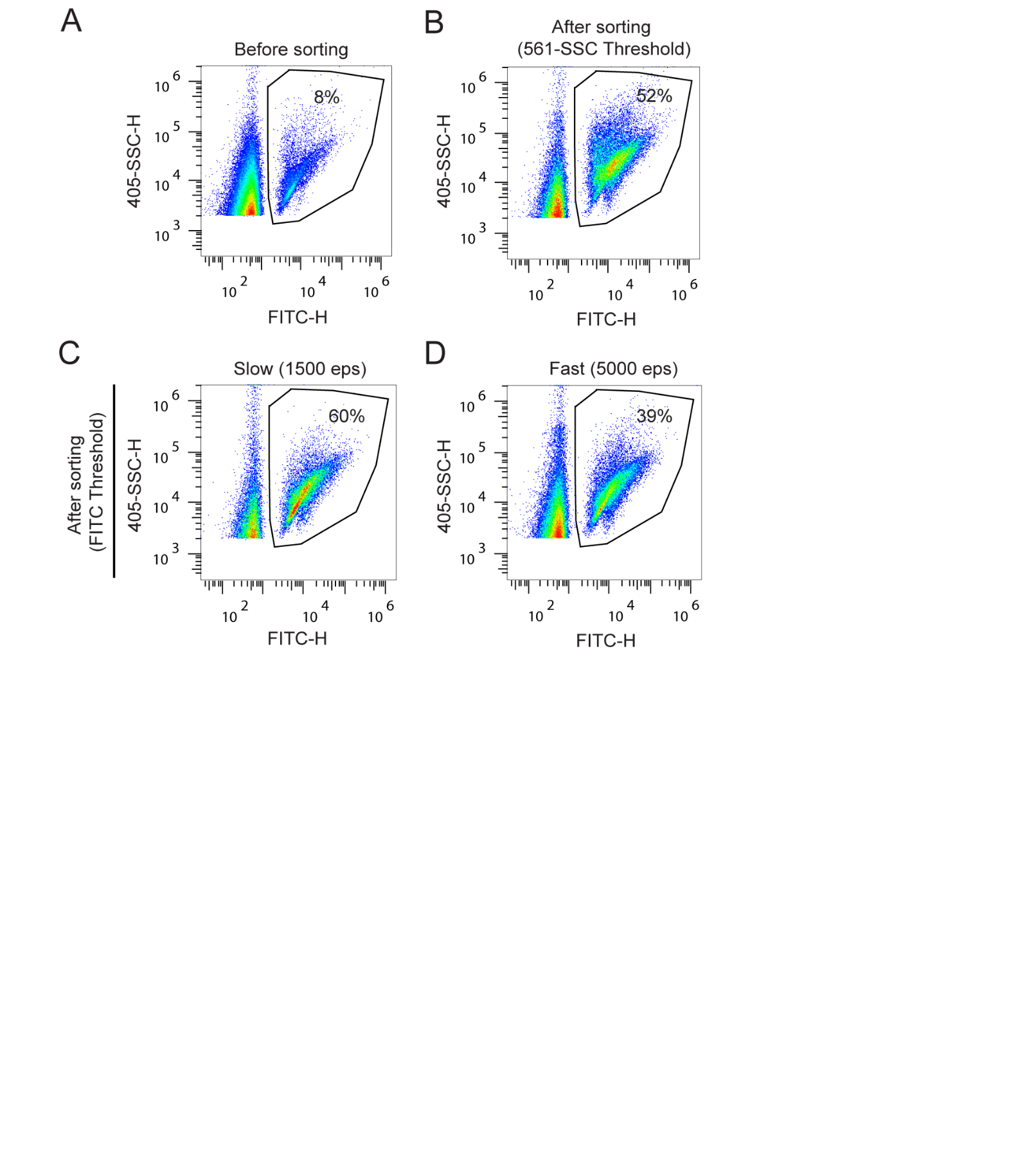


**Supporting Figure S3.** Purity of the sorting by FACS and optimization of the speed of sorting. (A-D) Dot plots (405-SSC-H vs FITC-H) of the measurements in high-sensitivity flow cytometry of a sample of organelles with nanoparticles before and after sorting. The sample of organelles with nanoparticles shown in Figure S1 was measured by high-sensitivity flow cytometry on a Cytoflex S before and after sorting. The percentage of organelles with nanoparticles before (A) and after (B) sorting was compared to check the quality of the sorting. When the threshold was applied in the yellow-red laser SSC-H (B)the purity of the sample increased around 6 times. A similar result was achieved with the same sample when the threshold for detection was applied in the FITC channel (~7 times) (C). With both settings (B&C) the sample was sorted using a speed of 1500 events per second. Instead, when sorting the sample with the threshold in the FITC channel but with a faster speed (5000 eps) (D), the quality of the sorting decreased to around a 5-fold increase. Based on these results, the organelles were sorted with FITC-H threshold at slow speed (1500 eps, C), because in these conditions all the detected events were sorted, whereas when sorting with yellow-red laser SSC-H threshold only a smaller percentage of fluorescence events was sorted (22%, see Supporting Figure S2B). Therefore, the sorting was faster when applying the FITC-H threshold, despite using lower speed to achieve higher purity.

**
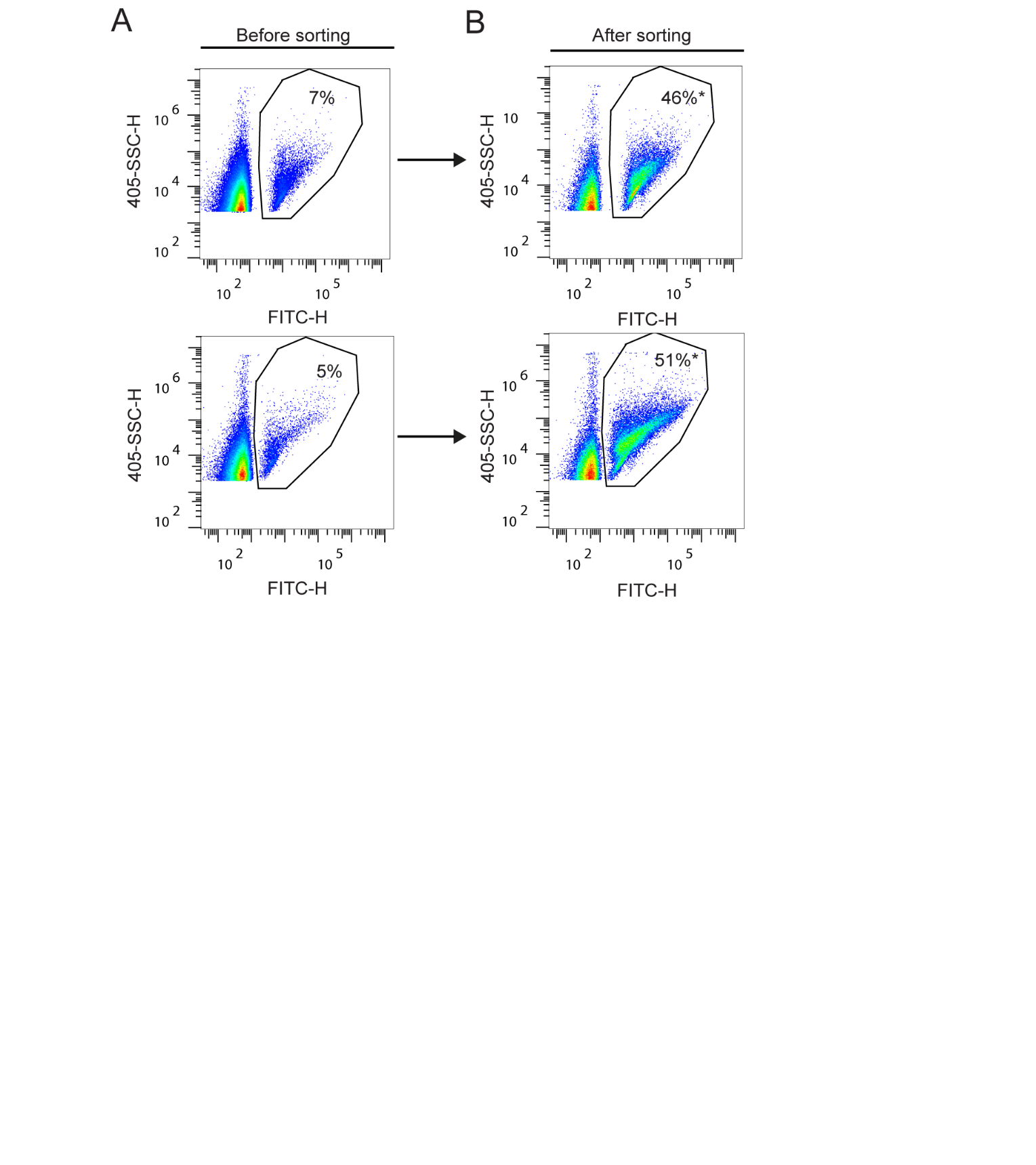
**

**Supporting Figure S4.** Quality of the sorting in the samples used for proteomics characterization. (A-B) Dot plots (405-SSC-H vs FITC-H) of the measurements in high-sensitivity flow cytometry of a sample of organelles with nanoparticles before and after sorting. Briefly, HeLa cells were incubated with yellow-green PS-COOH 100 nm nanoparticles for 30 minutes at 100 µg/ml and the organelles were extracted and sorted with the optimized settings as described in Methods. Before (A) and after (B) sorting, the percentage of fluorescent organelles was checked (7 and 5%, respectively before sorting and 46% and 56%, after). The sorting resulted in approximately 9-10 fold increase in purity of the population of interest. In the dot plots in B, the percentage of fluorescent organelles was corrected for the background. Briefly, the number of background events can be estimated by measuring a sample of PBS with the same flow rate using the same settings. We previously showed that when measuring at 1500-3000 eps, as used here for the sorting, the number of background events is minimal (less than 2% of the total events recorded when measuring an organelle sample with the same settings).^[2]^ However, after sorting the sample is diluted to approximately 500 eps. In these conditions, the contribution of events from the background is higher, and this affects the calculation of the percentage of fluorescent organelles in the sample. To account for that, 30 µl of a sample only containing PBS and of the sorted sample were measured at the same flow rate. Then, the events counted in the PBS sample were subtracted from the total events in the sorted sample, and the new percentage was calculated after this correction. The same data are shown in Figure 1D without this background correction.

| **Surfactant** | **Percentage of keratin proteins** | **Proteins detected** |
| --- | --- | --- |
| SDC | >20% | 184 ± 225 |
| SDS | >90% | 22 ± 7 |
| Urea | >90% | 35 ± 13 |
| Rapigest | >5% | 321 ± 15 |

**Supporting Table S1.** Proteins detected by mass spectrometry in sorted samples recovered after ultracentrifugation by using different surfactants. The table shows the percentage of the total intensity corresponding to keratin proteins (here used as an estimation of contamination) and the total number of proteins detected in the sorted organelle samples, after filtering out the potential contaminants database from MaxQuant as described in the Methods. A sample of HeLa cell organelles was prepared as described in Methods and a total of 40,000,000 fluorescent events were sorted for each condition. The sample was ultracentrifuged at 80,000 g for 1 hour and resuspended in 50 µl sodium deoxycholate (SDC) 2.5%, sodium dodecyl sulfate (SDS) 1%, Urea 8 M or Rapigest 0.2% for 2 hours. Next, the samples were prepared for proteomics as described in the Methods, each following slightly different protocols depending on the surfactant used for the recovery, as detailed below. At least 2 independent experiments were performed for each conditions. The results showed that only when using Rapigest a high number of proteins was identified, while keeping a low level of contamination. Recovery with SDC resulted in too high variability in the number of proteins identified, with some attempts giving similar results as Rapigest and others with only very few proteins detected. When using SDS or urea, instead, a very low number of proteins was identified and the contamination was very high (>90%). Both SDS and urea protocols require additional purification steps prior to mass spectrometry using solid-phase-extraction columns, and this may result in peptide loss, likely explaining these outcomes. On the contrary no solid phase column was required when using SDC and Rapigest. .Based on these results, recovery with Rapigest was used for all the sorted samples and controls for proteomics characterization.


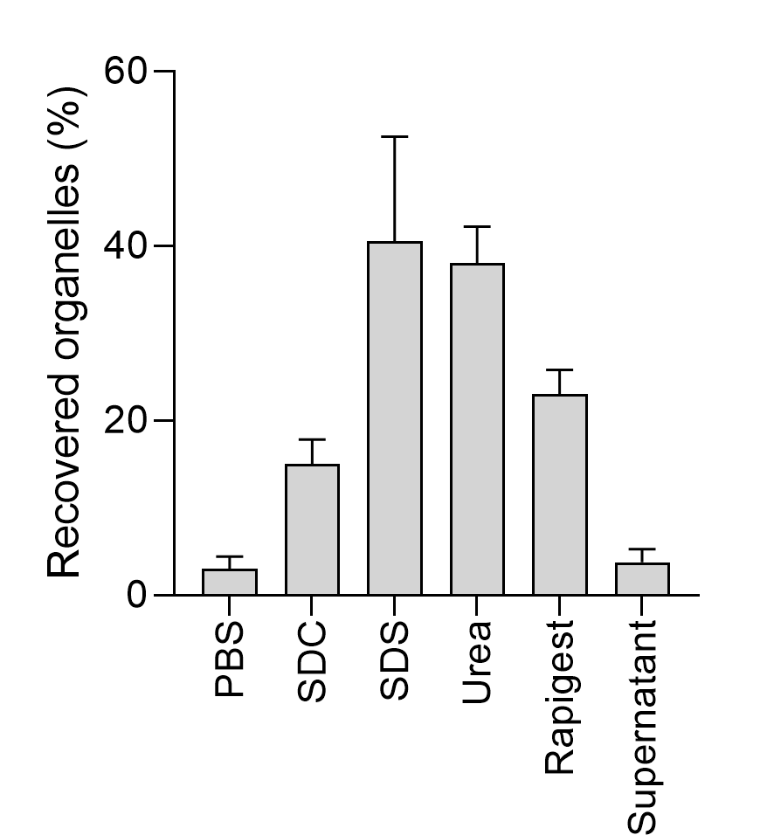


**Supporting Figure S5.** Recovery of the sorted organelles after ultracentrifugation. Bar plot showing the fluorescent organelles counted before and after ultracentrifugation using different surfactants compatibles with sample preparation for proteomics. After sorting the fluorescent organelles, the sample was loaded in the Cytoflex S flow cytometer and 30 µl were measured. Next the sample was ultracentrifuged at 80,000 g for 1 hour and resuspended in 50 µl of PBS, or 2.5% sodium deoxycholate (SDC), 1% sodium dodecyl sulfate (SDS), 8 M Urea or 0.2% Rapigest for 2 hours. Measurements of the supernatant confirmed that the organelles were correctly pelleted (not shown). The results showed that it was very difficult to recover the organelles in the pellet without the use of any surfactant. The best recovery was achieved when SDS or urea were used. The results are the average and standard deviation of the percentage of recovered organelles in at least 2 independent experiments for each condition.


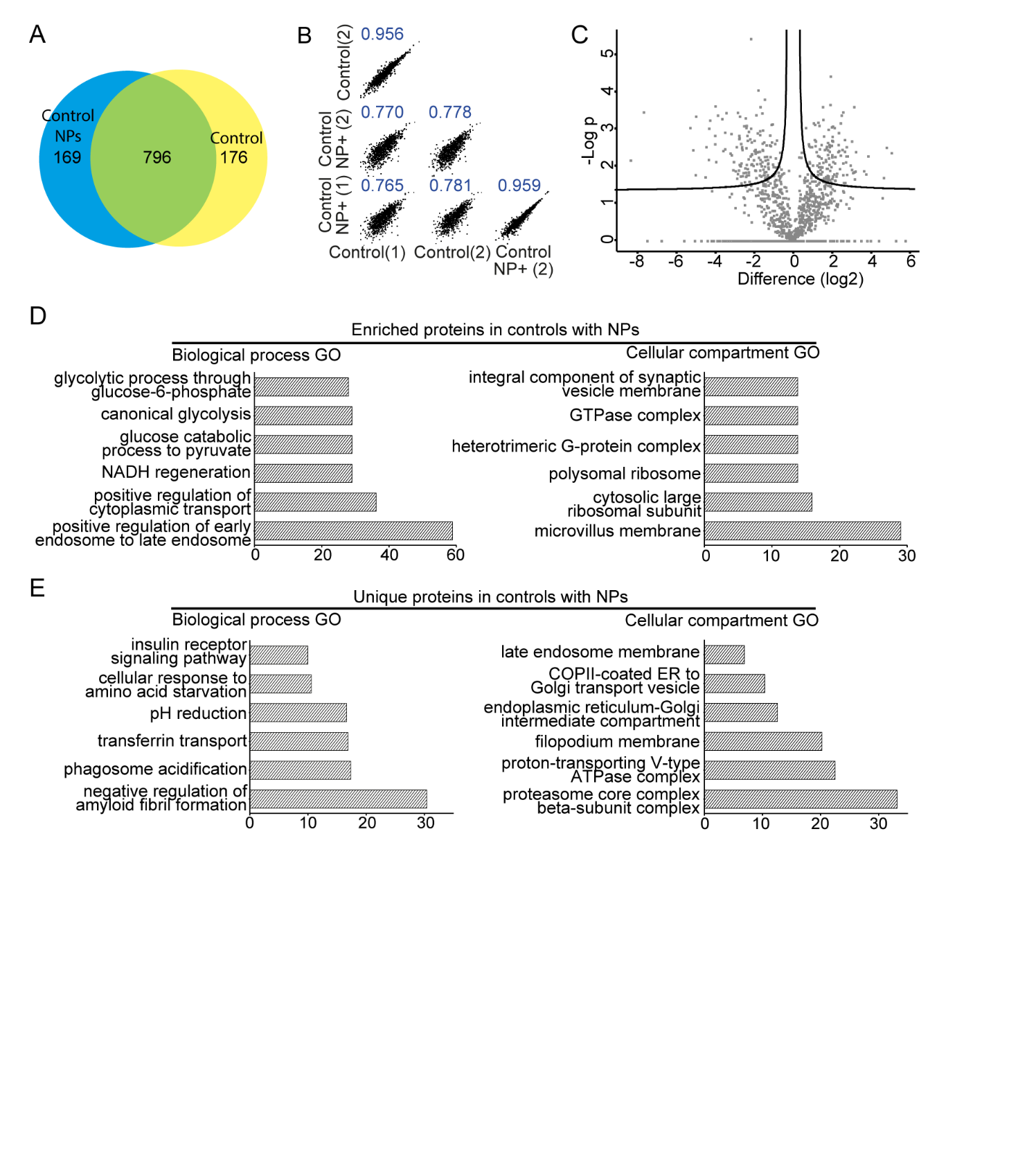


**Supporting Figure S6.** Proteomic characterization of the unsorted organelles recovered from untreated cells (control) and from cells incubated with nanoparticles (control NPs). (A) Venn diagram comparing the total number of proteins identified in the controls from cells incubated with nanoparticles and untreated cells. (B) Scatter plot comparing the iBAQ of the proteins identified in common for each replicate. The Pearson correlation is showed for each pair. Very high correlation (~0.96) was observed between replicate samples, confirming the reproducibility in the procedures for organelle extraction. (C) Volcano plot showing the enriched proteins in unsorted organelles from cells exposed to nanoparticles in respect to the unsorted organelles from untreated cells. A t-test was performed to determine the significance of the difference in iBAQ of the proteins. The solid line corresponds to a 0.01 FDR. (D) Statistically overrepresented biological process and cellular compartment gene ontology (GO) groups of the significantly enriched proteins in the unsorted organelles from cells incubated with nanoparticles. (E) Statistically overrepresented biological process and cellular compartment gene ontology (GO) groups of the unique proteins only present in the unsorted organelles from cells incubated with nanoparticles (169 proteins, Venn diagram A). (D-E) The gene ontology groups were sorted for fold enrichment and a threshold of minimum 3 proteins per group and p<0.01 was applied. A Fisher’s exact test was performed to determine the p-values. The results showed that in the organelles recovered from cells incubated with nanoparticles many of the common proteins were enriched (D) and at the same time several unique proteins were identified (E) which were not detected in the organelles recovered from untreated cells.


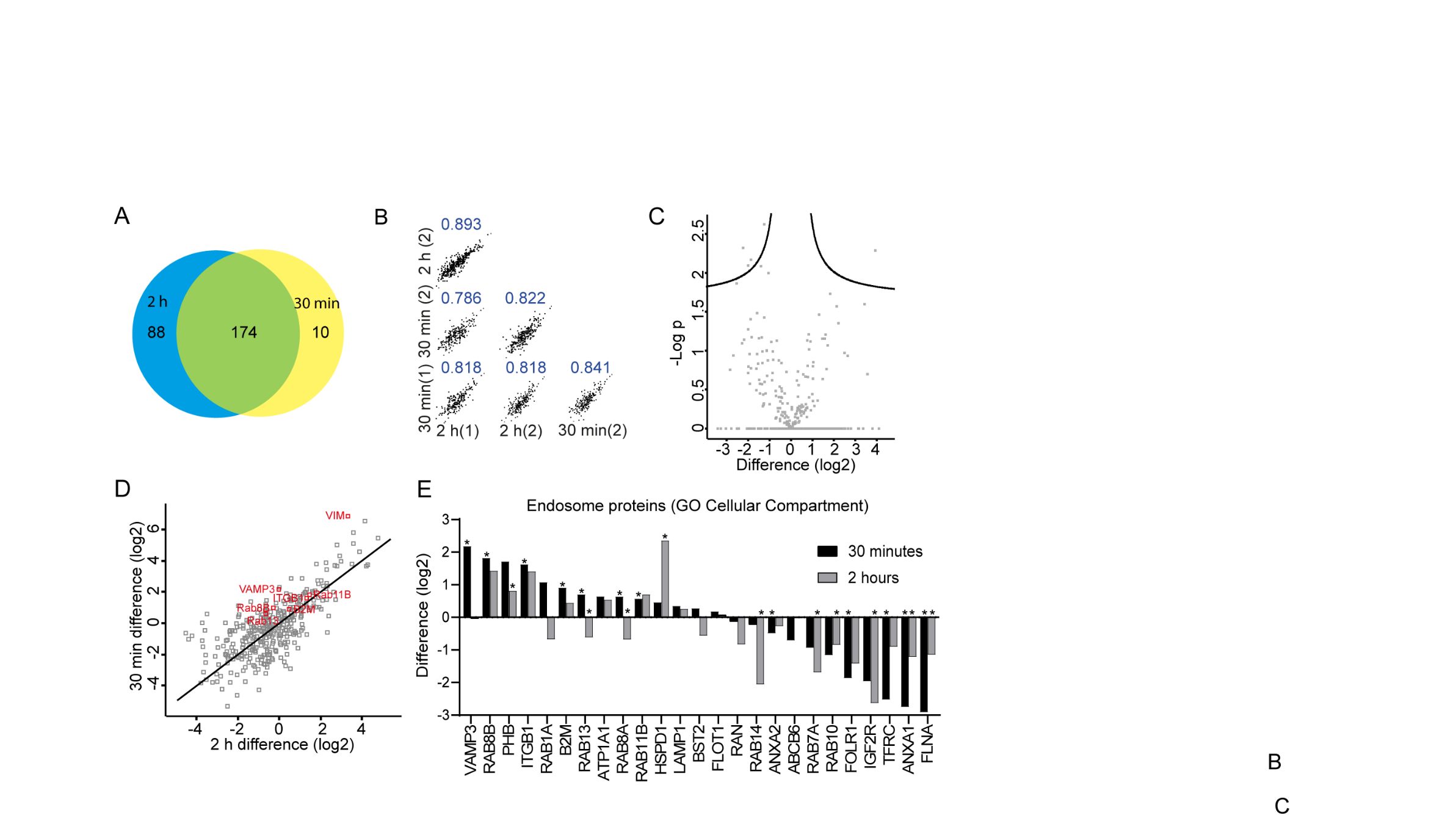


**Supporting Figure S7.** Comparison of the composition of the sorted organelles with nanoparticles recovered after 30 minute or 2 hour incubation. (A) Venn diagram comparing the total number of proteins identified in the sorted organelles recovered after 30 minute and 2 hour uptake. The results suggested that the composition of the organelles in which the nanoparticles are found after 30 minutes or 2 hours is almost the same (B) Scatter plot comparing the iBAQ of the proteins identified in each replicate. (C) Volcano plot showing the enriched proteins in the 30 minute organelles in respect to the 2 hour samples. A t-test was performed to determine the significance of the difference in iBAQ of the proteins. The dotted line corresponds to a 0.05 FDR. (D) Scatter plot comparing the protein enrichment of the sorted samples after 30 minute or 2 hour incubation, in respect to the proteins in the unsorted organelles from untreated cells (not exposed to nanoparticles). The regression line shows the points where the enrichment was the same for both samples (x=y). The proteins in red were the proteins selected for further validation. All of them were more enriched in the 30 minute sample than in the 2 hour sample. (E) Comparison of the enrichment of the endosomal proteins in the samples sorted after 30 minutes or 2 hours. All the proteins from the gene ontology group “Endosome (Cellular compartment)” which were present in the sorted samples are shown. The bar plot shows the enrichment of the endosomal proteins after sorting in respect to the organelles recovered after the same incubation time before sorting. The asterisks show the proteins significantly enriched. A t-test with FDR 0.05 was performed to determine significance. Almost no enrichment was observed when comparing the composition of the organelles in which nanoparticles were found after 30 minutes or 2 hours (C), however a slightly higher enrichment was observed in the 30 minute sample, in respect to the composition of the organelles from untreated cells (D) and for the endosomal proteins in respect to the organelles obtained at the same exposure time before sorting (E).

**
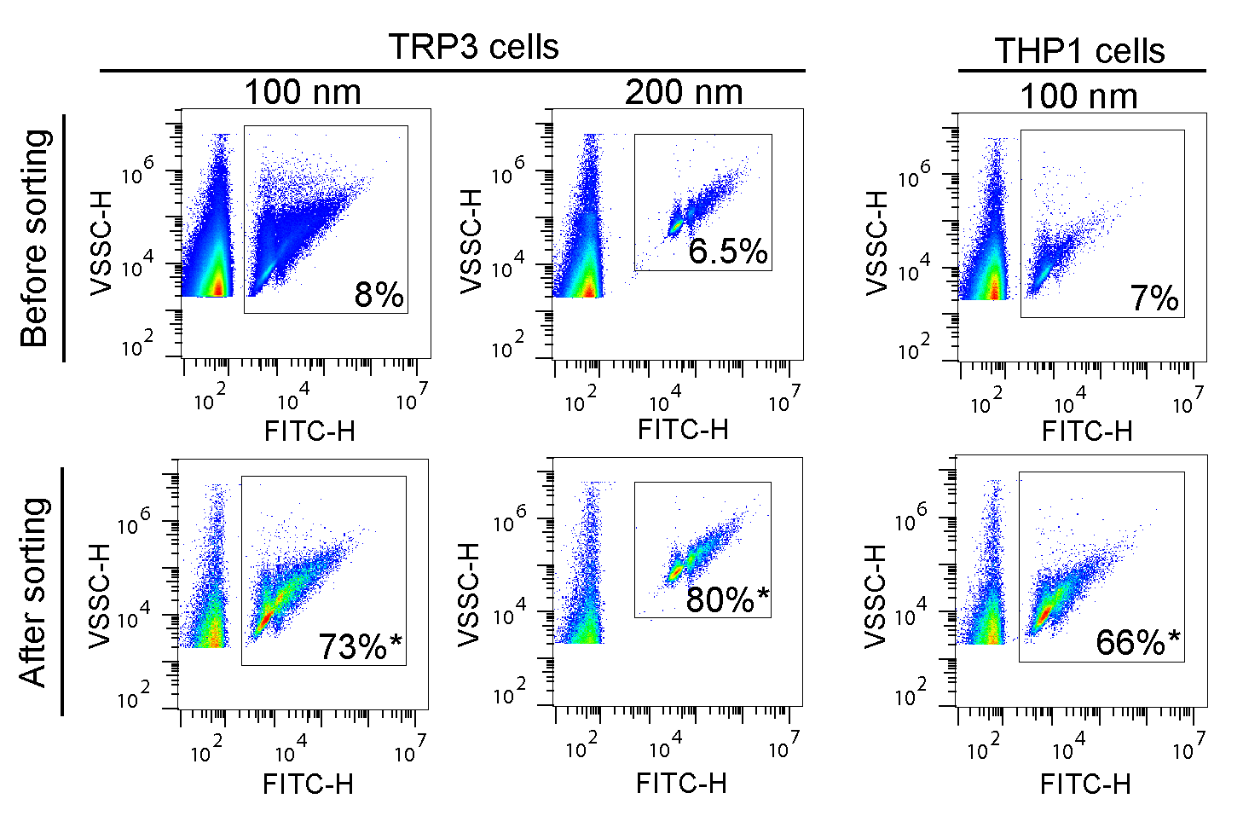
**

**Supporting Figure S8.**  Organelle sorting for other nanoparticle and cell types. Human TRP3 liver sinusoidal cells and THP1 monocytes were incubated with yellow-green fluorescent PS-COOH nanoparticles of 100 and 200 nm at a concentration of 100 and 200 µg/ml, respectively, for 30 minutes in cell culture medium supplemented with 40 mg/ml human serum (see Supporting Methods for details). Then, cells were lysed, and after cell fractionation, the organelles with nanoparticles were sorted by FACS as described in the Methods. The results show high sensitivity flow cytometry dot plots of Violet SSC and nanoparticle fluorescence (FITC) of the recovered organelles before sorting (upper graphs) and after sorting (lower graphs) by FACS. The population of organelles containing the fluorescent nanoparticles can be easily separated from the rest of the organelles. The results confirmed increased purity after sorting (roughly 10x), hence demonstrating that the method can be easily transferred to other cell types and to other nanoparticles, as well as that it can be used with nanoparticle dispersions at high serum concentrations closer to physiological conditions. By sorting the organelles recovered from different cells and after incubation with different types of nanoparticles and by further analyzing their composition by proteomic analysis, the method allows to characterize how the organelles in which nanoparticles are internalized and trafficked vary in different cell types and depending on nanoparticle properties (e.g. in this example nanoparticle size).


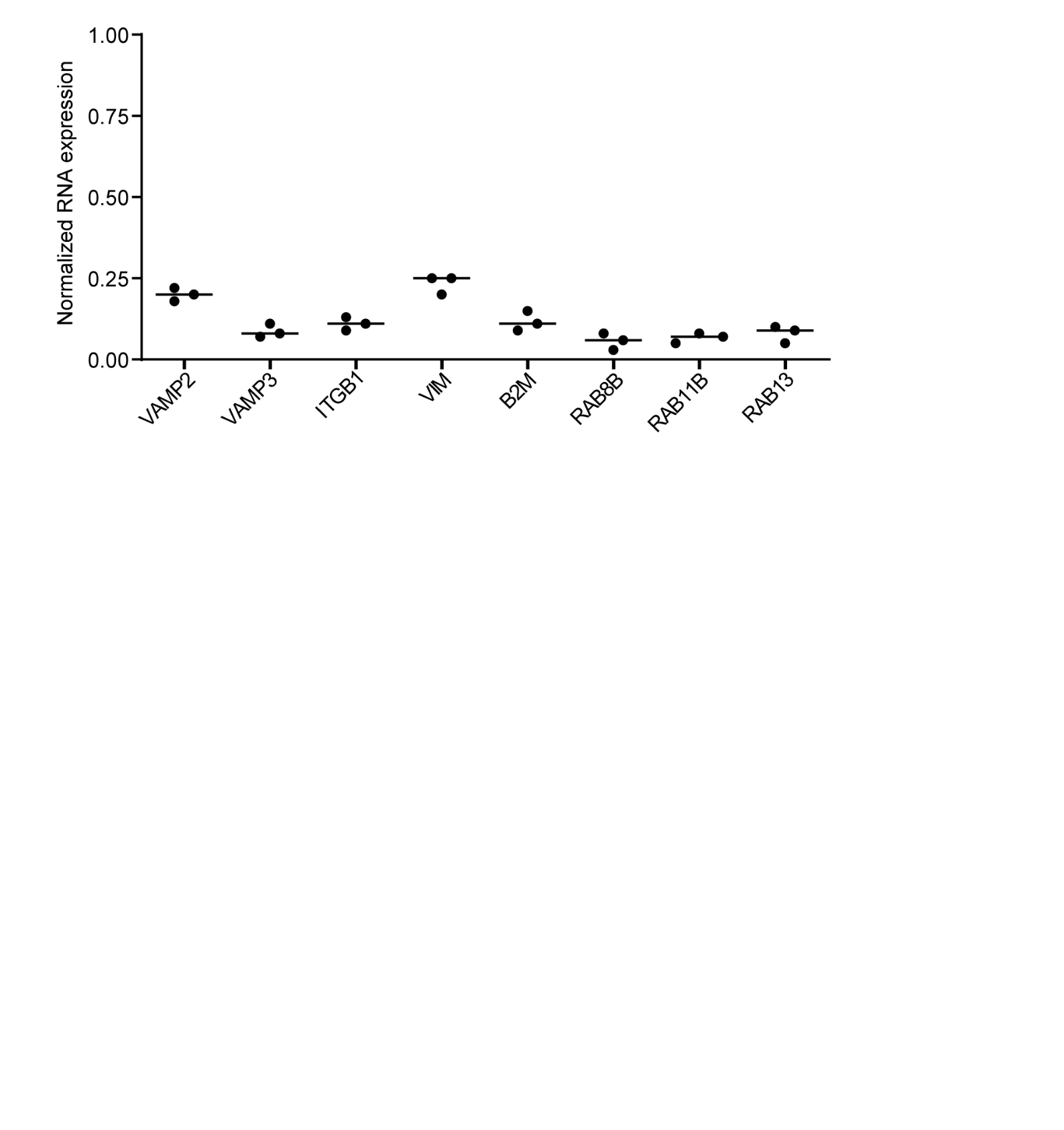


**Supporting Figure S9.** RNA expression levels after silencing of the protein targets selected for validation. Briefly, the expression of a panel of the proteins identified by organelle proteomic (VAMP2, VAMP3, ITGB1, VIM, B2M, RAB8B, RAB11B and RAB13) was silenced by RNA interference. Cells silenced with a scramble siRNA were also included as a control. Next the RNA was extracted from the cells and RT-PCR was performed for the different targets as indicated in the Methods. The plot shows the RNA expression in the silenced cells normalized by the expression in the control cells silenced with scramble siRNA. All the targets were silenced efficiently (> 90% for most targets, and ~75% for VAMP2 and VIM). The results in 3 replicate samples are shown and the solid line shows the average of the 3 replicates.


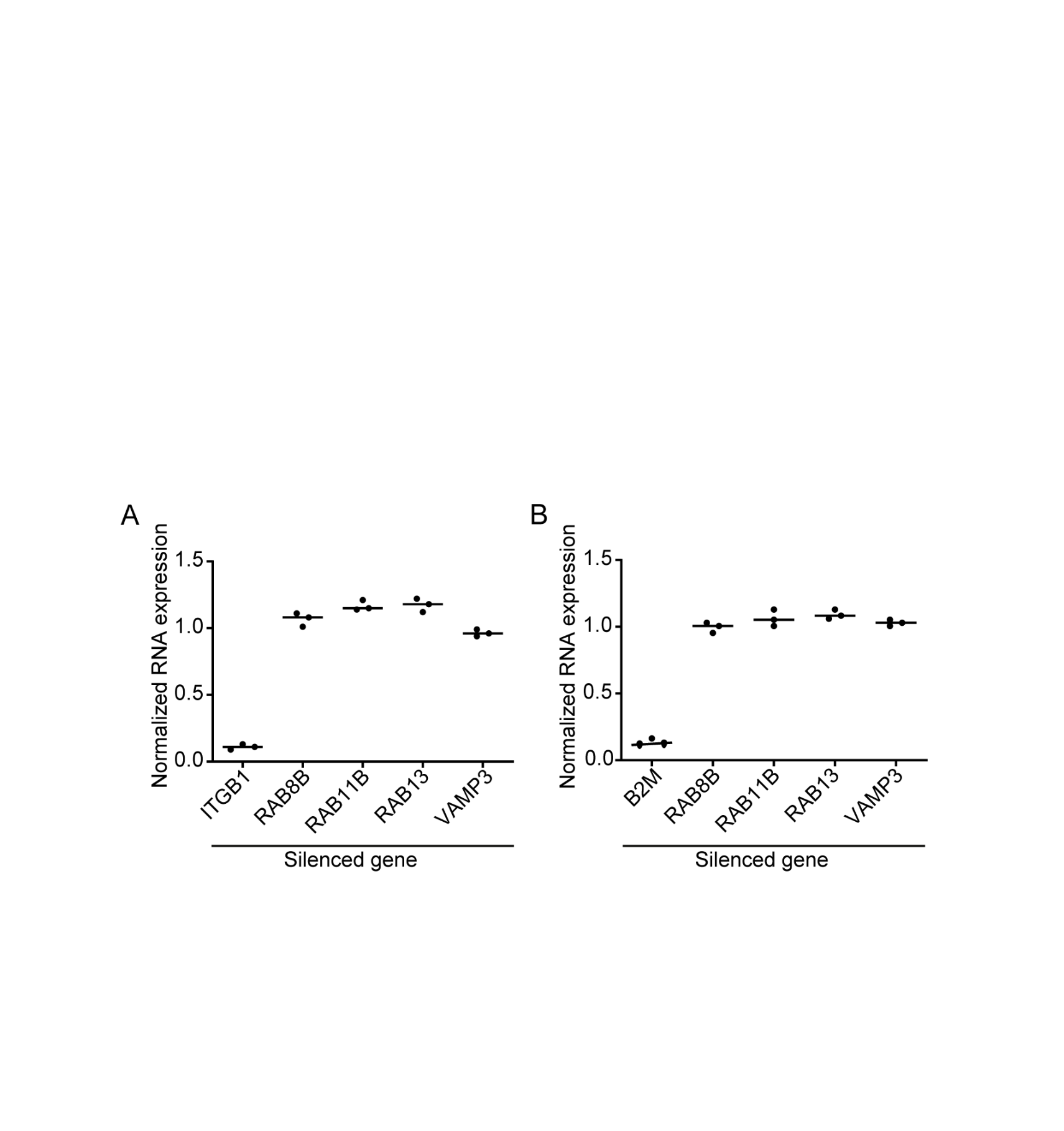


**Supporting Figure S10.** RNA expression levels of ITGB1 and B2M after silencing the expression of some of the identified proteins. Briefly, the expression of a panel of proteins, including ITGB1, B2M, RAB8B, RAB11B, RAB13 and VAMP3 was silenced in HeLa cells. Next the expression of ITGB1 (A) or B2M (B) was determined in the silenced cells in order to identify eventual indirect effects on their expression. . The plots show ITGB1 (A) or B2M (B) RNA expression in the silenced samples, normalized by the expression in control cells silenced with a scramble siRNA. A total of 3 replicate samples were made for each target, and the solid line shows the average of the 3 replicates. The results showed that silencing ITGB1 (A) or B2M (B) expression did not affect the mRNA expression levels of the other tested targets.


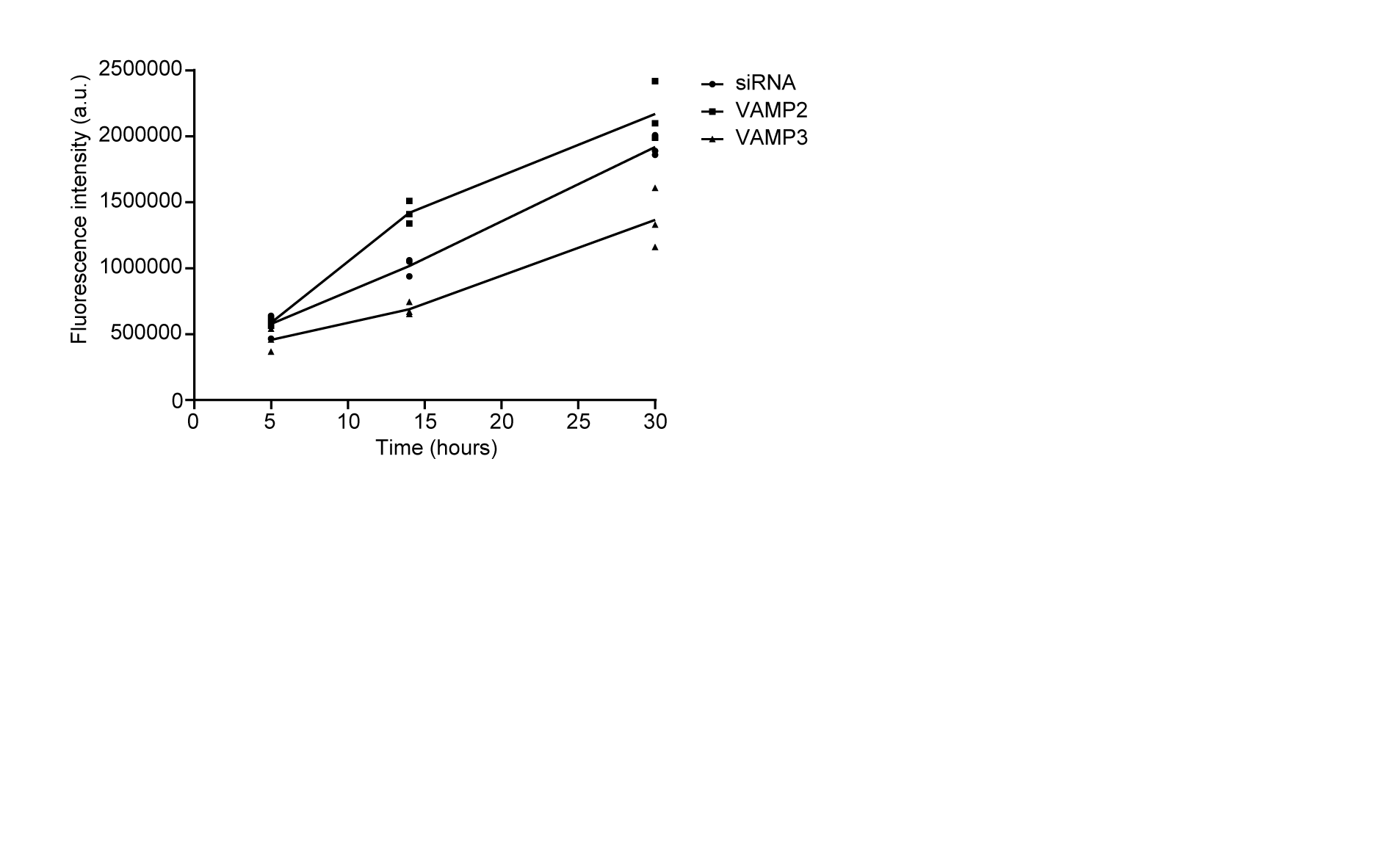


**Supporting Figure S11.** Nanoparticle uptake kinetics in Hela cells after silencing VAMP2 or VAMP3 expression. Briefly, HeLa cells were silenced for VAMP2, VAMP3, or a scramble siRNA as a control (siRNA). Next, the cells were incubated with 25 µg/ml 100 nm yellow-green PS-COOH nanoparticles and uptake by cells was measured by flow cytometry after different incubation times (5, 14 and 30 hours). The results obtained in 3 replicate samples are shown, together with a line that passes through their average.


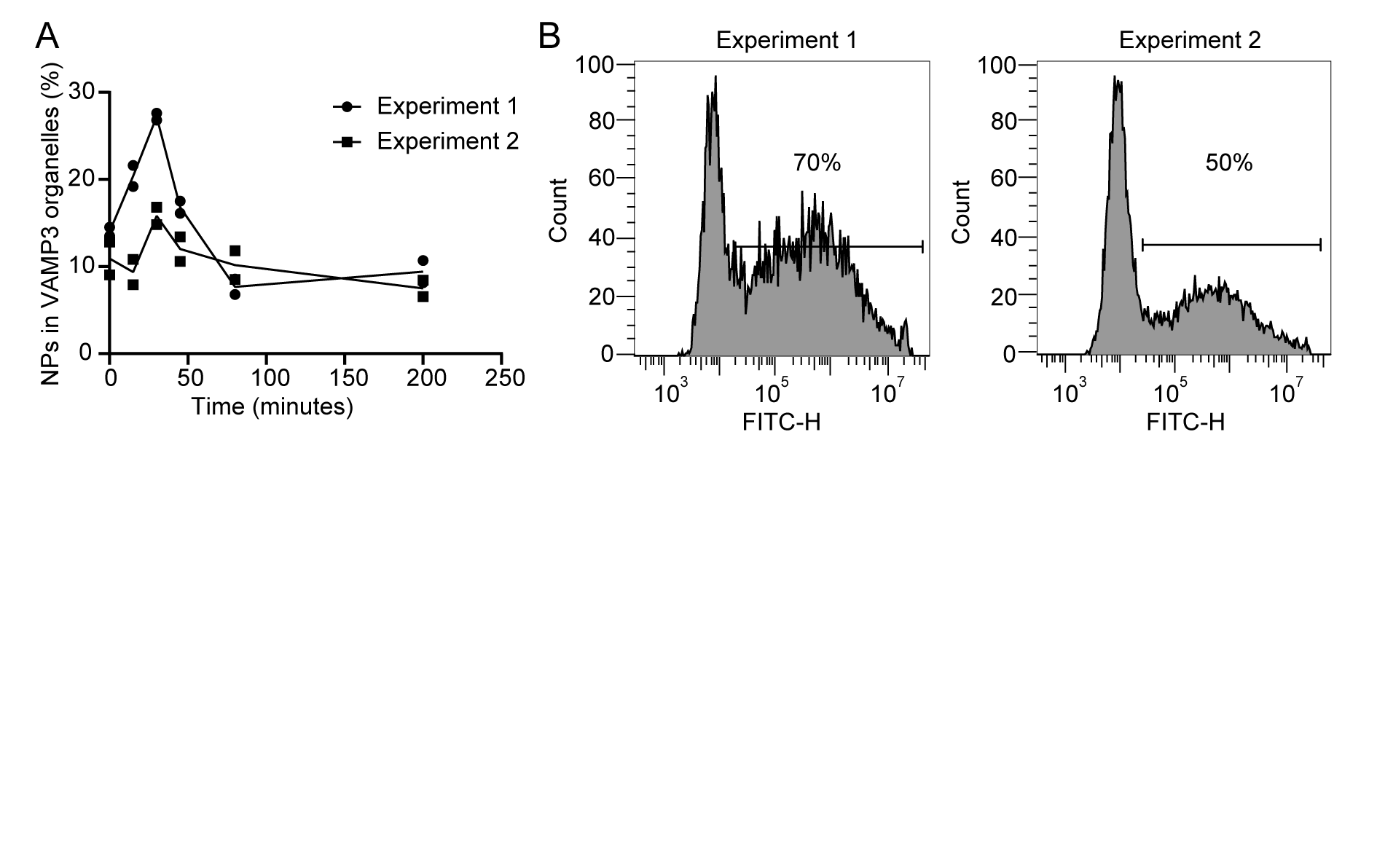


**Supporting Figure S12.** Colocalization of nanoparticles in VAMP3^+^ organelles. Briefly, HeLa cells were transfected with a construct to express eGFP-labelled VAMP3 as indicated in the Methods. Next, cells were incubated with 100 µg/ml 100 nm red PS-COOH nanoparticles for 15 minutes (pulse), then the nanoparticle dispersion was removed and cells further grown in nanoparticle-free medium and the organelles were extracted at different times (0, 15, 30, 45, 80 and 200 minute after nanoparticle removal (chase)). Panel A shows the percentage of nanoparticles in VAMP3-positive organelles at different chase times in 2 independent experiments. The results of two replicate samples are shown, together with a line that passes through their average. The same results are shown in Figure 4A after correction for transfection efficiency. (B) A small amount of transfected cells was measured prior to cell lysis in order to determine the transfection efficiency of the eGFP-VAMP3 plasmid in the two experiments. In panel B, the corresponding cell fluorescence distribution are shown. The results showed that the transfection efficiencies were 70 and 50 % for the experiments 1 and 2 respectively. The results of panel A are shown again in Figure 4A after normalization by these values in order to compare the kinetics of nanoparticle colocalization in VAMP3-positive compartments.


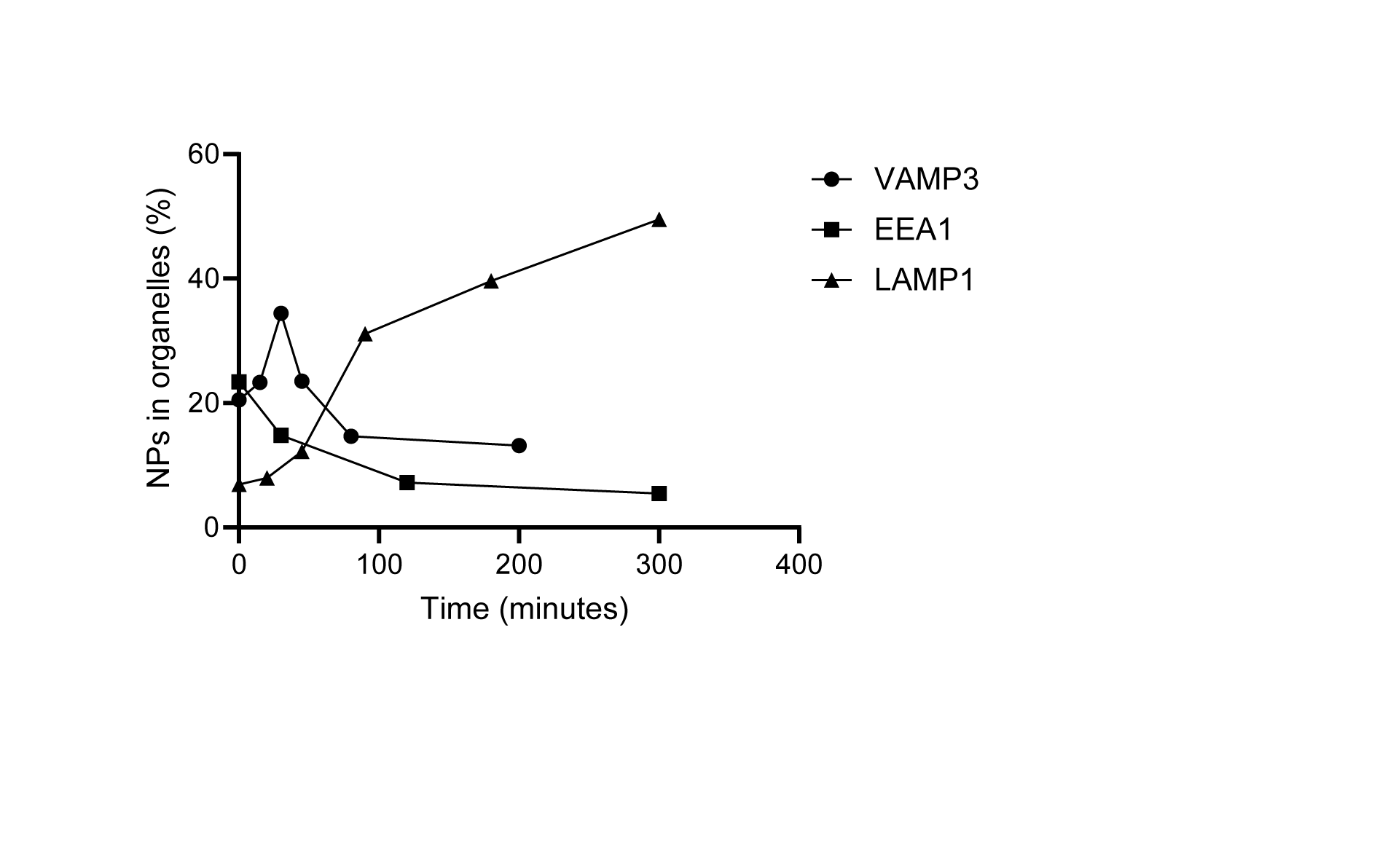


**Supporting Figure S13.** Intracellular trafficking kinetics of 100 nm in different compartments. The colocalization of the 100 nm nanoparticles in different organelles at different times after a short incubation time (pulse) is shown. The plot shows the VAMP3 colocalization results from Figure 4A together with the results obtained in the same way in a previous study,^[2]^ here reproduced for comparison, on the colocalization of the same nanoparticles in EEA1-stained early-endosomes and LAMP1-stained lysosomes. All the experiments were performed in HeLa cells incubated with 100 nm PS-COOH nanoparticles at 100 µg/ml for 15 minutes (VAMP3) or 30 minutes (LAMP1 and EEA1). The results are the average obtained from 2 independent experiments for each target (with 2 replicate samples at each time). The overlap of the colocalization results at different times shows that these nanoparticles first transit in early endosomes, then pass into VAMP3-compartments and later arrive in the lysosomes were they accumulate over time.


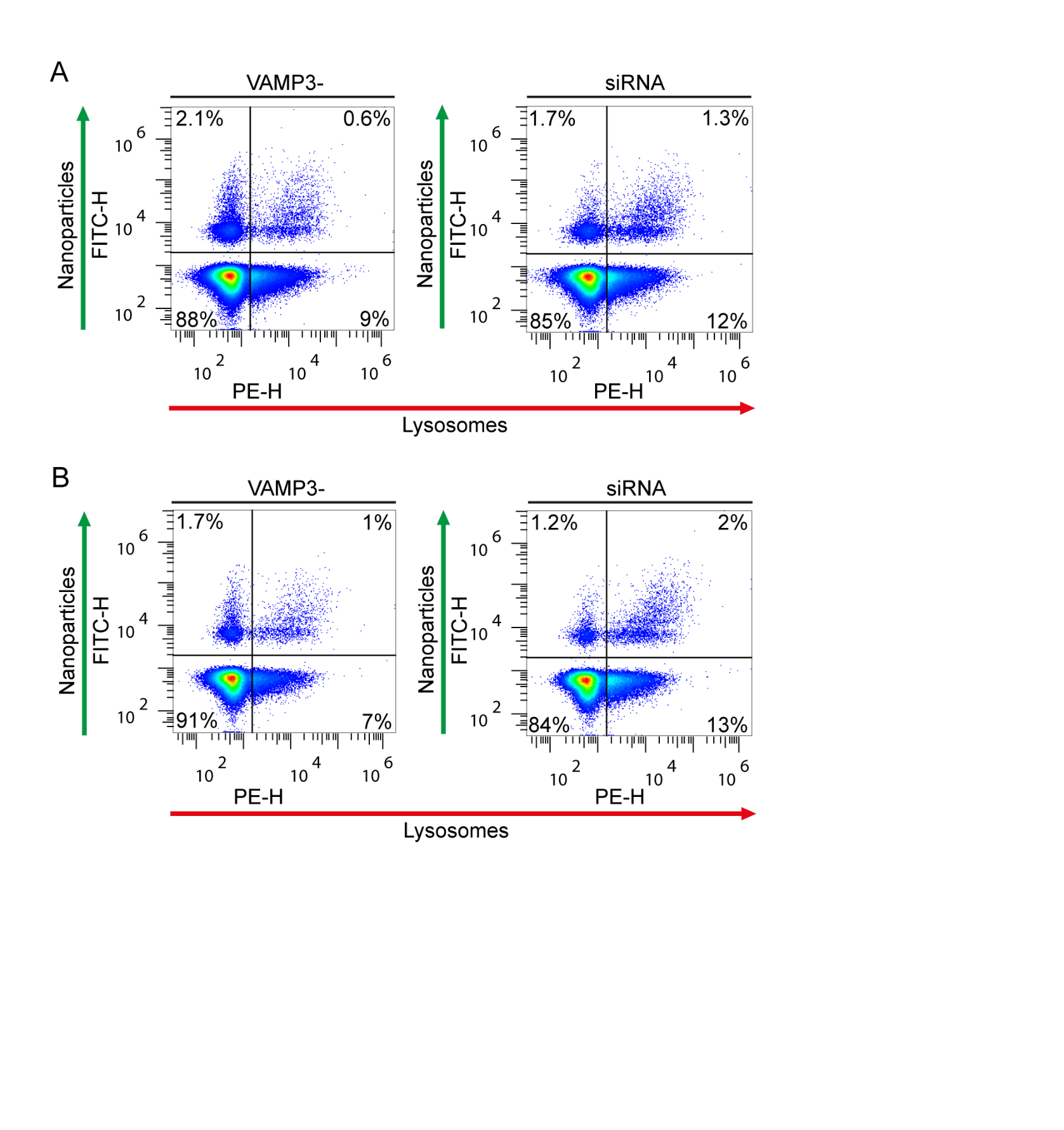


**Supporting Figure S14.** Colocalization of 100 nm nanoparticles in the lysosomes after silencing VAMP3 expression. Briefly, HeLa cells were silenced for VAMP3, and scramble siRNA was used as a control. Next, the cells were incubated with 100 nm yellow-green PS-COOH nanoparticles at 100 µg/ml for 3 hours. Then, the nanoparticle dispersion was removed and cells were further grown for 10 hours in nanoparticle-free medium (chase) (A). Alternatively, cells were incubated with 25 µg/ml 100 nm PS-COOH for 15 hours uptake. Afterwards, the organelles were extracted and immunostained with a LAMP1-Cy3 antibody to detect the lysosomes as indicated in the Methods. The dot plots (A-B) show the fluorescence of the nanoparticles in the FITC channel versus the fluorescence of the lysosomes in the PE channel. In the top right quadrant, the lysosomes with nanoparticles can be detected. The results showed that in both experiments, around 50 % reduction in the colocalization of the nanoparticles with the lysosomes could be observed after silencing VAMP3 expression (A-B). The colocalization percentage obtained in these results is shown in Figure 4C.

**References**

1. Parent, R.; Durantel, D.; Lahlali, T.; Sallé, A.; Plissonnier, M.-L.; DaCosta, D.; Lesca, G.; Zoulim, F.; Marion, M.-J.; Bartosch, B., An immortalized human liver endothelial sinusoidal cell line for the study of the pathobiology of the liver endothelium, *Biochemical and biophysical research communications* **2014,** *450* (1), 7-12. DOI https://doi.org/10.1016/j.bbrc.2014.05.038.

2. Garcia Romeu, H.; Deville, S.; Salvati, A., Time- and Space-Resolved Flow-Cytometry of Cell Organelles to Quantify Nanoparticle Uptake and Intracellular Trafficking by Cells, *Small* **2021,** *17* (34), 2100887. DOI https://doi.org/10.1002/smll.202100887.
